# Colliding Challenges: An Analysis of SARS-CoV-2 Infection in Patients with Pulmonary Tuberculosis versus SARS-CoV-2 Infection Alone

**DOI:** 10.3390/medicina60050823

**Published:** 2024-05-16

**Authors:** Camil Mihuta, Adriana Socaci, Patricia Hogea, Emanuela Tudorache, Monica Simina Mihuta, Cristian Oancea

**Affiliations:** 1 Department of Doctoral Studies, “Victor Babes” University of Medicine and Pharmacy, 300041 Timisoara, Romania; camil.mihuta@umft.ro; 2 Clinical Hospital for Infectious Diseases and Pneumology “Dr. Victor Babes”, 300041 Timisoara, Romania; hogea.patricia@umft.ro (P.H.); emanuela.tudorache@umft.ro (E.T.); oancea@umft.ro (C.O.); 3 Department of Biology and Life Sciences, Faculty of Medicine, “Vasile Goldis” Western University of Arad, 310025 Arad, Romania; 4 Center for Research and Innovation in Precision Medicine of Respiratory Diseases (CRIPMRD), “Victor Babes” University of Medicine and Pharmacy, 300041 Timisoara, Romania; 5 Department of Pulmonology, “Victor Babes” University of Medicine and Pharmacy, 300041 Timisoara, Romania; 6 Center of Molecular Research in Nephrology and Vascular Disease, Faculty of Medicine, “Victor Babes” University of Medicine and Pharmacy, 300041 Timisoara, Romania; simina.mihuta@umft.ro

**Keywords:** COVID19 outcomes, immunocompromised in *SARS-CoV-2* infection, *SARS-CoV-2* and tuberculosis co-infection, pulmonary tuberculosis

## Abstract

*Background and Objectives*: The concurrent occurrence of tuberculosis and COVID-19 coinfection poses significant clinical complexities, warranting a nuanced approach to diagnosis, management, and patient care. *Materials and Methods*: A retrospective, cross-sectional study was conducted on two groups: one comprising 32 patients with pulmonary TB (PTB) and COVID-19 co-infection, and one including 100 patients with COVID-19 alone. Data was collected from medical records, including patient history, clinical parameters, laboratory, imaging results, and patient outcome. *Results*: A lower BMI emerges as a significant marker suggesting underlying PTB in patients with *SARS-CoV-2* co-infection. Type 2 diabetes mellitus increases the risk of death in PTB-*SARS-CoV-2* co-infection. Co-infected patients show lymphocytopenia and higher neutrophil levels, CRP, transaminases, and D-dimer levels. Elevated CRP and ALT levels are linked to increased co-infection likelihood. Certain parameters like SpO2, CRP, ALT, AST, and D-dimer effectively differentiate between co-infected and COVID-19 patients. Platelet-to-lymphocyte ratio is notably higher in co-infected individuals. Lesion severity on imaging is significantly associated with co-infection, highlighting imaging’s diagnostic importance. Longer hospital stays are linked to co-infection but not significantly to death risk. *Conclusions*: Certain clinical and biological factors may serve as potential indicators of PTB co-infection in patients with *SARS-CoV-2*.

## 1. Introduction

Tuberculosis (TB) is an infectious disease with profound implications for human health. *Mycobacterium tuberculosis* primarily invades the lungs, but it can also disseminate to other organs and tissues, including the intestines, liver, lymph nodes, skin, brain, and various systems such as musculoskeletal and reproductive [1].

The year 2020 is likely to be etched in memory as the “COVID-19 year”, marked by the emergence of the severe acute respiratory syndrome coronavirus 2 (*SARS-CoV-2*) responsible for the pandemic. While COVID-19 continues to dominate both scientific literature and the media, it is crucial not to overlook other communicable diseases, including TB [2,3,4,5,6]. Since 2019, the genetic makeup of the *SARS-CoV-2* reference sequence has undergone alterations, leading to speculations about the emergence of variants capable of evading immune responses and resisting treatments and vaccines [7,8,9]. Phylogenetic analysis indicated that the Romanian epidemic commenced with numerous introduction events from various European countries, succeeded by localized transmission [10]. Roughly all variants ultimately have circulated on Romanian territory, but the most prominent at the time of our study (the initial months of the pandemic) were variants pertaining to lineage B, subtype B.1.5 and B.1.1 [10,11]. These variants produced a high variability in symptomatology and severity. Diabetes and arterial hypertension are comorbidities which were shown to be associated with a more severe disease form [11]. 

Amid the World Health Organization’s (WHO) declaration of COVID-19 as a Public Health Emergency of International Concern, considerable attention has been devoted to exploring the potential interactions between *SARS-CoV-2* and TB infection. The prevailing view, shared by the WHO and specialized scientific sources, suggests that the COVID-19 pandemic could exacerbate the global TB epidemic. This anticipated worsening is attributed to various factors, including additional strains on health systems from COVID-19, leading to the weakening of National TB programs. The potential biological effects of the interaction between the two infections are also emphasized, reminiscent of the historical concept of a ‘cursed duet’, previously applied to TB and HIV [7,8,9,10,11,12,13,14,15,16].

Unlike COVID-19, TB is an ancient menace that has plagued humanity for millennia, with 25% of the global population harboring latent *Mycobacterium tuberculosis* infection, and within this group, 5–15% may progress to develop active TB during their lifetime. The risk of reactivation varies both geographically and individually [12,17]. Hence, the convergence of COVID-19 and TB poses grave challenges, impacting TB diagnosis, treatment, and control programs [18,19]. Individuals with active TB are often immunocompromised, making them more vulnerable to COVID-19’s severe effects. Consequently, the synergistic relationship between TB, HIV, and COVID-19 has created a global syndemic, underscoring the urgent need for comprehensive research on TB/COVID-19 co-infection [20]. The two pathogens, *Mycobacterium tuberculosis* and *HIV*, can mutually potentiate each other, accelerating the deterioration of immune functions. Each of these diseases holds significance and can notably impact lung function through distinctive cytokine storms, immunosuppression, and respiratory failure. Reported co-infections of *SARS-CoV-2* with *HIV* and *Mycobacterium tuberculosis* can alter their pathogenesis and disease advancement. Individuals with pulmonary tuberculosis and *HIV*/AIDS might exhibit heightened susceptibility to *SARS-CoV-2* infection, potentially resulting in lethal synergistic effects and increased disease severity [21]. Moreover, worldwide, it is estimated that between 10 to 25% of TB infections occur extrapulmonarily, infecting virtually any organ, thus definitive diagnosis usually requires invasive procedures and complex imaging examinations, as symptoms can be very heterogeneous [22].

Both lung TB and COVID-19 share similarities in their airborne transmission, lung-centric effects, symptoms, and social determinants. However, their pathogenesis differs significantly, suggesting that understanding their interactions could inform new prevention and treatment strategies for TB/COVID-19 co-infection. Common clinical manifestations of COVID-19 include fever, respiratory symptoms such as dispnoea, tachypnea, cough, and even hemoptysis, along with less severe symptoms such as fatigue, headache, myalgia, and gastrointestinal symptoms like vomiting and diarrhea. Prolonged cough is a primary symptom for both lung TB and COVID-19 [20,23,24,25]. While limited information exists on the risk and severity of concurrent TB and COVID-19, previous studies suggest the potential exacerbation of TB in co-infection with certain viruses, such as measles. TB patients face a potential risk of co-infection with *SARS-CoV-2* and vice-versa [17,25,26].

With regard to biomarkers, COVID-19 and TB present elevated levels of C-reactive protein, D-Dimer, and interleukin 6, but also alterations such as leukopenia, neutrophilia or even platelet dysfunctions. These shared clinical parameters and underlying immunological reactions imply that co-infection may not only complicate diagnostic processes but also lead to a potentially fatal convergence in immunopathogenesis [27]. Macrostructural pulmonary changes caused by TB, such as fibrosis and bronchial obstructions, compromise lung function and defense mechanisms, potentially worsening COVID-19 outcomes. These insights underscore the importance of addressing TB/COVID-19 co-infection comprehensively [28].

Hence, the need for additional research specifically addressing the TB-COVID-19 co-infection is evident. This study aims to better understand and mitigate the impact of this novel pathogenic combination. We assessed differences in several clinical, biological and imagistic markers in order to better understand the impact of moderate and severe forms of *SARS-CoV-2* infection on patients with recently diagnosed lung TB.

## 2. Materials and Methods

The present retrospective, cross-sectional, randomized study involved 132 adult patients aged 39 to 81, hospitalized at the Victor Babeș Hospital of Infectious Diseases and Pneumoftiziology from Timișoara. We analyzed data from March to June 2020. The design of the study was based on the comparison of two groups of patients, one with lung tuberculosis and *SARS-CoV-2* co-infection (*n* = 32), and a control group including age-matched patients with a diagnosis of only moderate or severe *SARS-CoV-2* infection (*n* = 100). The main objective was to determine the impact of *SARS-CoV-2* on an already tarred organism due to TB. 

*Inclusion criteria:*

□ pulmonary TB (PTB) diagnosed 1 month prior to the confirmation of *SARS-CoV-2* infection at most, through solid or liquid cultures (Gene-Xpert) in the TB ambulatory service from Timișoara [29];
□ moderate or severe *SARS-CoV-2* forms of infection at the moment of hospital admittance, confirmed by nasopharyngeal exudate RT-PCR analysis in an acreditted laboratory. A moderate form was considered that in which the individual was exhibiting signs of lower respiratory disease during clinical evaluation or imaging, with oxygen saturation measured by pulse oximetry SpO2 ≥ 94% on room air.

A severe form is characterized by an SpO2 < 94% on room air, a respiratory rate exceeding 30 breaths/min, or lung infiltrates exceeding 50% [30].

□ normal renal function (normal GFR, creatinine and urea levels) [31];
□ BCG vaccination completed in all participants [32].

*Exclusion criteria:*

□ extrapulmonary TB, including pleural TB efussions, TB lymphadenopaties, miliar TB, osteoarticular TB, intestinal TB, urogenital TB, meningitis TB, and other forms.
□ overweight (BMI = 25 to 30 kg/m^2^) or obesity (BMI ≥ 30 kg/m^2^) [33];
□ pre-existing severe or uncontrolled arterial hypertension [34];
□ lung cancer [35] or other neoplasies [36];
□ idiopathic lung fibrosis [37,38];
□ pre-existing advanced chronic heart failure [39];
□ hepatic, renal, or digestive chronic conditions that may result in weight loss and *HIV* infection

The Ethics Council for Scientific Research at the Victor Babeș University of Medicine and Pharmacy Timisoara granted approval for the study, which adheres to the principles of the Helsinki Declaration (04/19 January 2021). Before enrollment in the study, informed consent was obtained from all patients after comprehensive explanations about the nature of the data analysis. 

### 2.1. Data Collection

The data was collected from personal medical files and included anamnestic information, clinical parameters and biological and imagistic investigations. 

The medical history revealed the BCG vaccination status. Comorbidities such as COPD and type 2 DM were noted. A part of the patients diagnosed with TB were observed to have had a history of TB prior to the present infection (all of them underwent appropriate therapy at the time and were deemed cured). The smoking status of each patient was determined: never smoked, smoker. Three social groups were analyzed as per employment: unemployed, employed, and retired.

The BMI was calculated using the standard formula Weight (kg)/Height^2^ (m^2^) [40]. The SpO2 (%) values obtained using calibrated pulse oximeters in the COVID-19 unit [41] were recorded from charts at two points: first, at the time of *SARS-CoV-2* infection diagnosis, and second, the lowest value recorded during hospitalization. Peripheral systolic and diastolic blood pressures (SBP, DBP, mmHg) were obtained from charts at diagnosis. The *SARS-CoV-2* symptoms noted in all patients were: fever, coughing, dispnoea, fatigue, abdominal pain, chest pain, myalgia, vomiting/nausea, diarrhea, headache, olfactory/taste disorders. The symptomatology was classified as more severe if any of these symptoms associated tachypnea (respiratory rate ≥ 30 breaths/min) [42].

We analyzed the following blood markers: C-reactive protein (CRP, mg/L), procalcitonin (PCT), aspartate aminotransferase (AST, U/L), alanine aminotransferase (ALT, U/L), lactate dehydrogenase (LDH, U/L), inteleukin-6 (IL-6, pg/mL), D-dimer levels (mg/L), neutrophil, lymphocyte and platelet count (/µL) [40]. We calculated the neutrophil to lymphocyte ratio (NLR) [43], the platelet to lymphocyte ratio (PLR) [44], and the systemic immuno-inflammatory index (SII), calculated as platelet count x NLR [45].

X-ray images were analyzed and the following lesions were noted at the moment of *SARS-CoV-2* infection:

□ unilateral pulmonary infiltrate, no cavities;
□ bilateral pulmonary infiltrates, no cavities;
□ unilateral pulmonary cavitary lesions;
□ bilateral pulmonary cavitary lesions.

We considered bilateral pulmonary cavitary lesions the most severe lesions.

An experienced radiology specialist (over 10-year experience) reviewed all CT scans completed at the confirmation of *SARS-CoV-2* infection

The chest CT interpretation focused on detecting lesions such as: ground-glass opacifications, consolidations, crazy paving pattern, linear opacities combined, air bronchogram sign, tree in bud [46], and cavitary lesions [47]. To quantify the extent of lung lesions, each of the five lung lobes was visually scored from 0 to 5, with the following classification:

0 points: no involvement;
1 point: less than 5% involvement;
2 points: 5–25% involvement;
3 points: 26–49% involvement;
4 points: 50–75% involvement;
5 points: more than 75% involvement.

The total chest CT involvement score was then determined by summing the individual scores from each lobe, yielding a range from 0 to 25. This approach provides a comprehensive view of the disease’s impact on the lungs [48,49].

Cavitary lesions were included in calculating the lung involvement score, but were also noted separately, as an aggravating factor suggestive for lung TB.

The number of total hospitalization days and the outcome (resolved-PCR converted or death) were also determined in each case.

### 2.2. Data Analysis

Data collection was conducted using Microsoft Excel for Microsoft 365 MSO (Version 2404 build 16.0.17531.20140) and statistical analyses were performed with MedCalc Statistical Software version 20.111 (MedCalc Software Ltd., Ostend, Belgium). The primary focus of the analysis was to examine the impact of *SARS-CoV-2* infection on patients with and without PTB co-infection. Clinical, imagistic and biologic parameters were assessed in comparison. Significance was determined by *p*-values below 0.05. To assess the normality of the data distribution, the Shapiro–Wilk test was employed. Subsequently, appropriate statistical tests were chosen based on the normality of the data: medians and the Mann–Whitney test were used for non-normal variables. The AUC-ROC analysis was employed in order to evaluate the significance of various parameters in discerning between the presence and absence of co-infection. Cut-off values for such discrimination were determined with the ultimate scope of highlighting the significance of certain parameters in evaluating these medical conditions. Logistic regressions were employed to identify independent predictors of co-infection and fatal outcome. The Fisher’s exact test was used to evaluate the associations between sets of binary data. 

## 3. Results

This study included 132 patients, divided in 2 study groups comprising 32 patients with PTB and *SARS-CoV-2* co-infection, and 100 patients with *SARS-CoV-2* infection alone. 

Out of the co-infected patients, 21 (65%) were male and 11 (34%) female. In the *SARS-CoV-2* group, 52 patients were male and 48 were female. The mean age in the co-infected group was 62.8 years, SD = 12.82, while the median age in the *SARS-CoV-2* group was 57 years; a significant difference in age between the two groups was not detected, *p* = 0.07 (Mann–Whitney test). 

**Figure 1:**
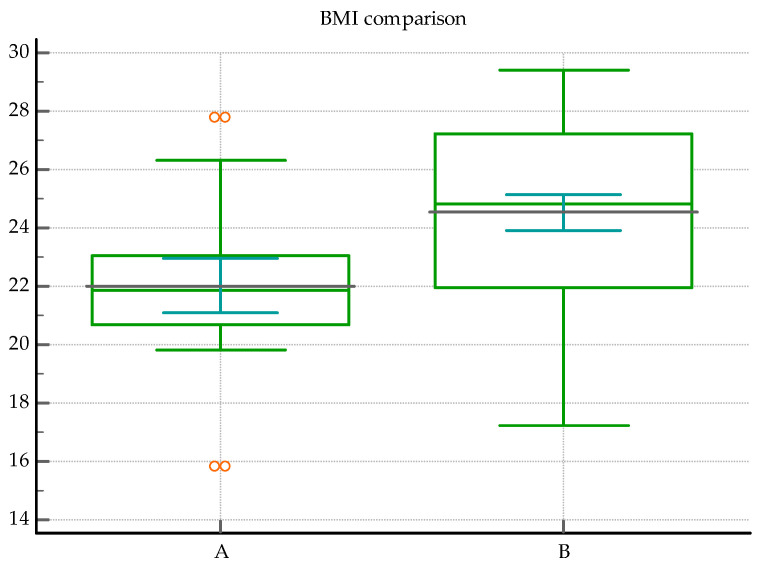
BMI comparison between the two groups: A = the co-infection group, B = the *SARS-CoV-2* group; *p* = 0.0002.

The BMI was significantly lower in the co-infection group, with a median value of 21.88 vs. 24.82, *p* = 0.0002 (Mann–Whitney test); see Figure 1. 

The comparison between co-infected and *SARS-CoV-2* infected patients showed that there are significant differences between the two groups in the majority of the analyzed parameters. The co-infected group revealed significantly lower SpO2 both at diagnosis, and with regard the lowest value registered, lower neutrophil and lymphocyte counts, and higher SBP levels, CRP, transaminase, and D-dimer levels. The chest CT involvement score was also significantly higher in the co-infection group. PLR was higher in the PTB-*SARS-CoV-2* group (Table 1).

A multiple parameter logistic regression was employed with the dependent variable being the presence/absence of PTB-*SARS-CoV-2* co-infection and the independent variables being clinical and anamnestic parameters: age, sex, BMI, employment status, associating COPD, type 2 DM, the severity of symptoms and the status of previously having TB. The BMI and the severity of symptoms emerged as significant predictors of PTB-*SARS-CoV-2* co-infection. The lower the BMI levels, the higher the odds of co-infection. Severe symptomatology is associated with a higher probability of co-infection. The rest of the variables were not included in the model. See Table 2.

The logistic regression employed for the paraclinical and biological parameters, with the dependent variable being the presence/absence of PTB-*SARS-CoV-2* co-infection and the independent variables being: SpO2 at diagnosis, lowest SpO2, SBP, DBP, CRP, LDH, PCT, IL-6, AST, ALT, D-dimer, neutrophil count, lymphocyte count, thrombocyte count, NLR, PLR, SII, and the chest CT involvement score. The model revealed as significant predictors (*p* < 0.0001) the SpO2 levels at diagnosis, LDH, ALT, neutrophil count, and the CT score (see Table 3). The higher the ALT levels and CT score, the higher the odds of co-infection, and the higher the SpO2 at diagnosis and LDH levels, the lower the probability of co-infection. The rest of the variables were not included in the model.

Further on, AUC-ROC analyses were employed in order to determine the significance of multiple parameters in discriminating between the presence and absence of PTB co-infection (Table 4).

**Figure 2:**
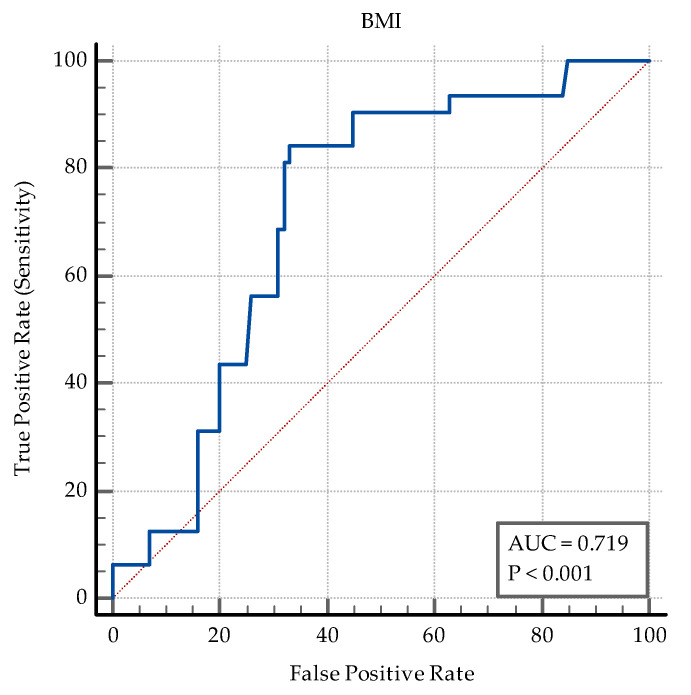
ROC curve for BMI.

**Figure A1:**
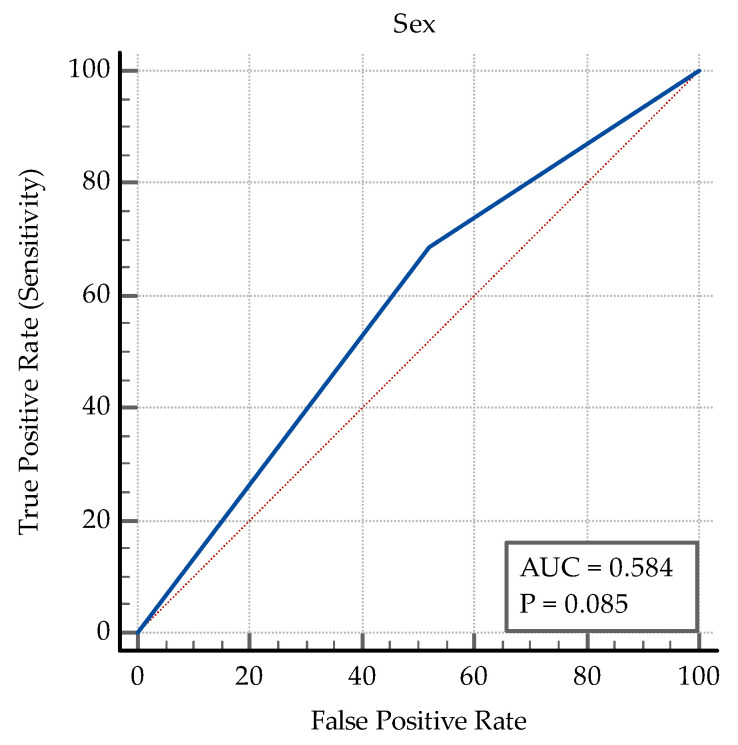
ROC curve for sex.

The AUC-ROC analysis showed that sex (Figure A1) and age are not reliable discriminators, *p* = 0.08, and *p* = 0.058, respectively. The analysis revealed that a cut-off value for the BMI ≤ 23.23 was significant for discriminating between the presence of PTB co-infection and its absence (*p* < 0.0001), see Figure 2 and Table 4. Hence, the smaller the value of BMI, the higher the probability of TB co-infection. 

**Figure 3:**
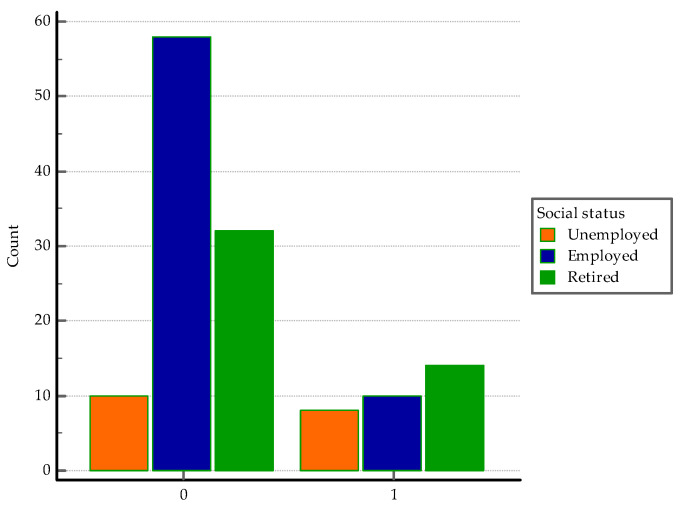
Distribution of cases according to the social status. 0 = *SARS-CoV-2* group, 1 = co-infection group.

**Figure A2:**
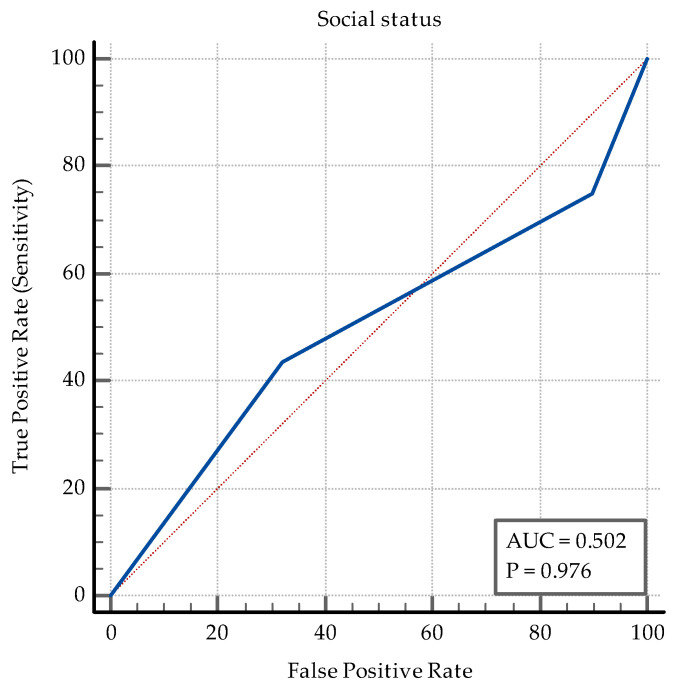
ROC curve for employment status.

With regard to employment status, in the co-infection group 25% were unemployed, 31.2% employed, and 43.8%, retired. In the *SARS-CoV-2* group, 10% were unemployed, 58% employed, and 32% retired. See Figure 3. The employment status does not seem to be significant for acquiring PTB-*SARS-CoV-2* co-infection, *p* = 0.97. Despite a relatively good sensibility, it revealed a very low specificity, according to the AUC-ROC analysis (Table 2, Figure A2).

**Figure A3:**
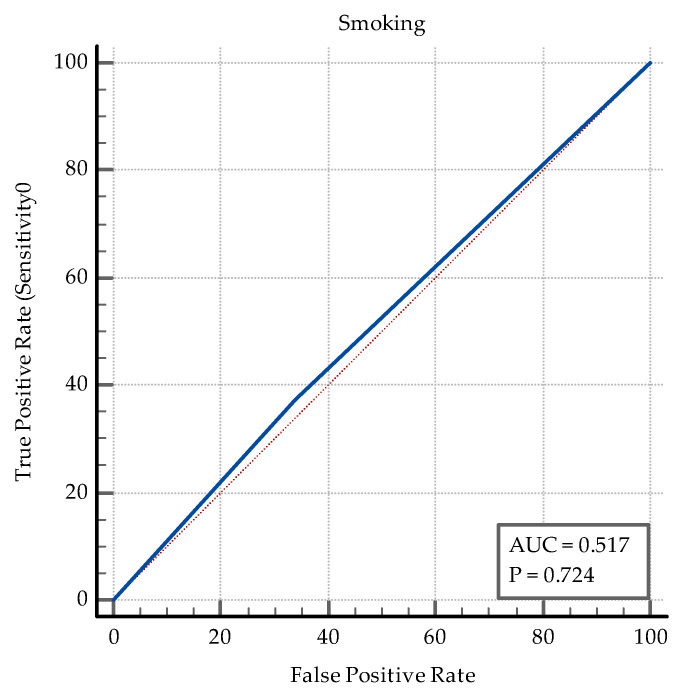
ROC curve for the status of smoking.

With regard to smoking, 37.5% patients in the co-infection group were smokers, while in the *SARS-CoV-2* group, 34%. The AUC-ROC analysis did not reveal a significant power of discrimination between the two groups of study, with relatively low sensitivity and specificity (Table 4, Figure A3).

**Figure A4:**
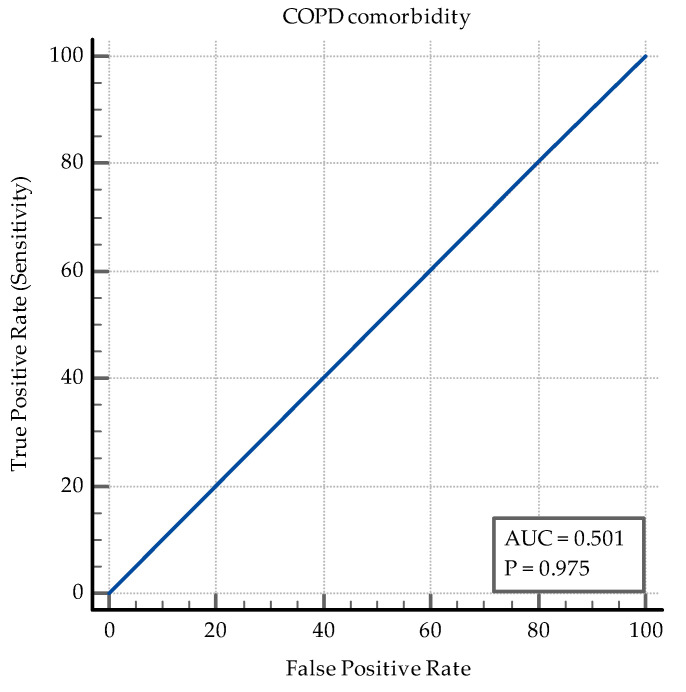
ROC curve for the association of COPD.

**Figure A5:**
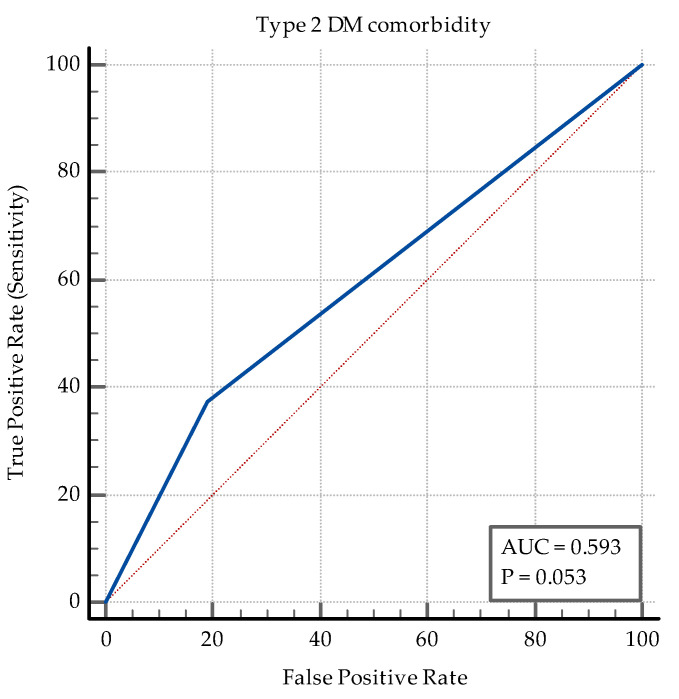
ROC curve for the association of type 2 DM.

In the co-infection group, 18.7% patients associated COPD, and 59.4% associated type 2 DM. In the *SARS-CoV-2* group, 19% also presented COPD, and 19%, diabetes. As for associating both conditions, 3 patients were detected in the co-infection group and 6 patients in the *SARS-CoV-2* group. Neither the association of COPD nor type 2 DM were significant factors for aquiring PTB-*SARS-CoV-2* co-infection, although in the case of diabetes, the analysis almost reached significance, with a *p* = 0.052 and a good specificity (Table 4, Figure A4 and Figure A5). 

**Figure 4:**
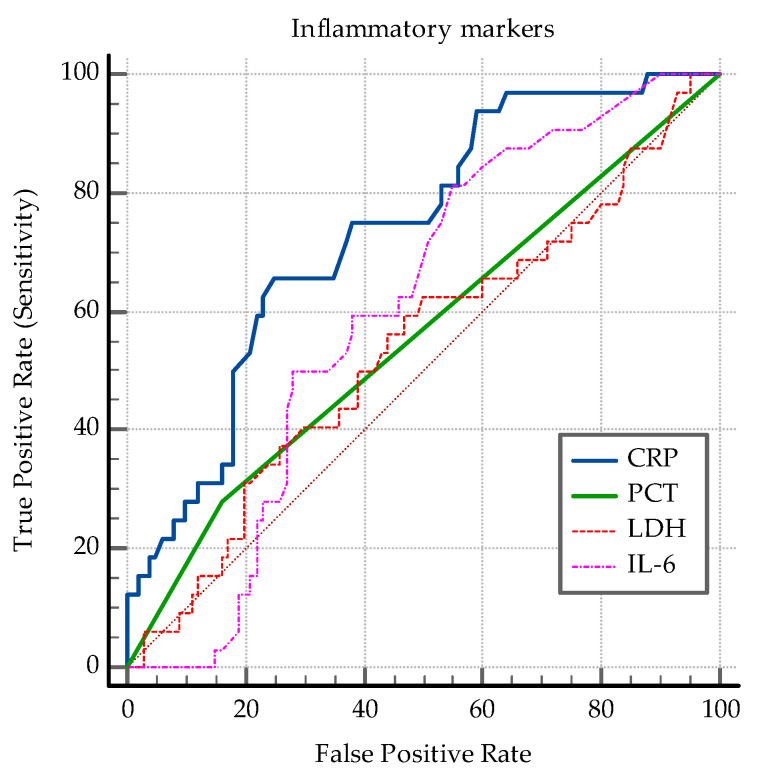
ROC curves for the inflammatory markers.

**Figure 5:**
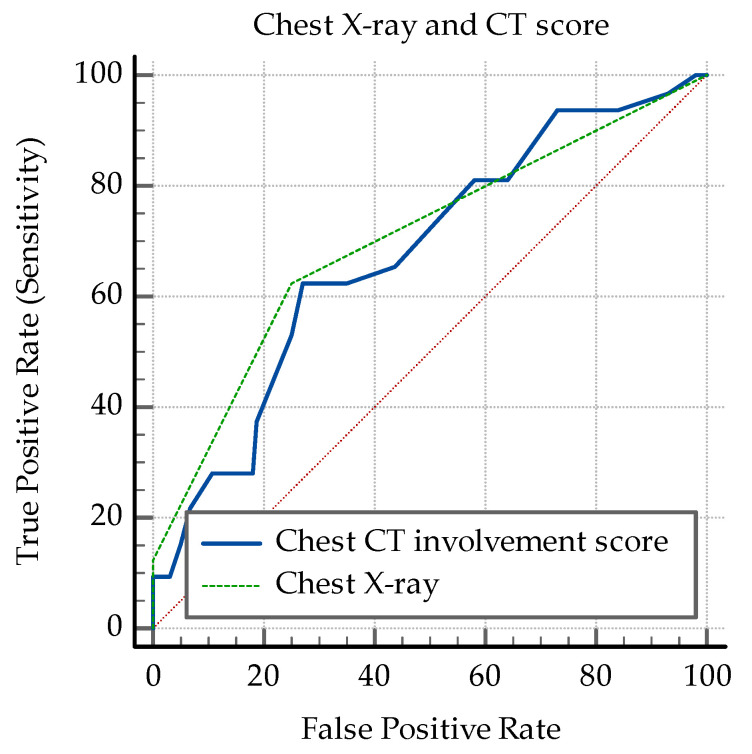
ROC curves for chest X-ray and CT involvement score.

**Figure A6:**
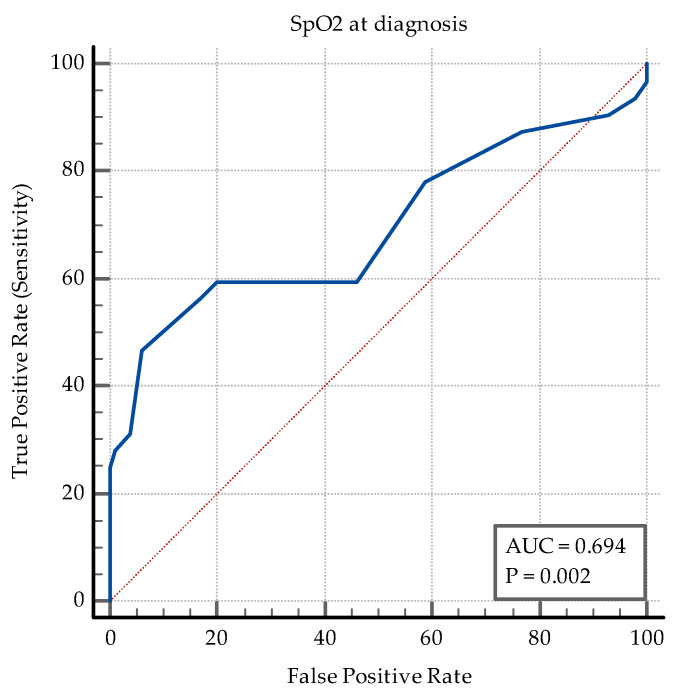
ROC curve for the values of SpO2 at diagnosis.

**Figure A7:**
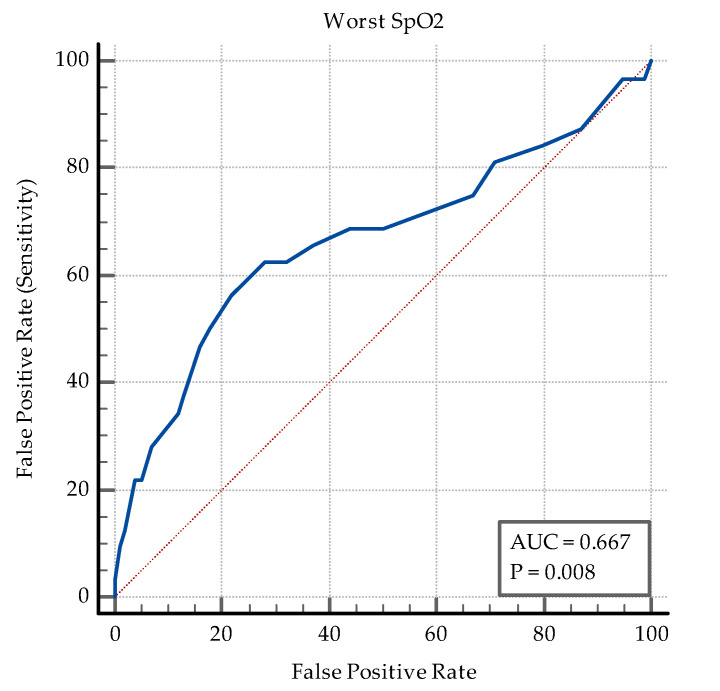
ROC curve for the lowest values of SpO2.

**Figure A8:**
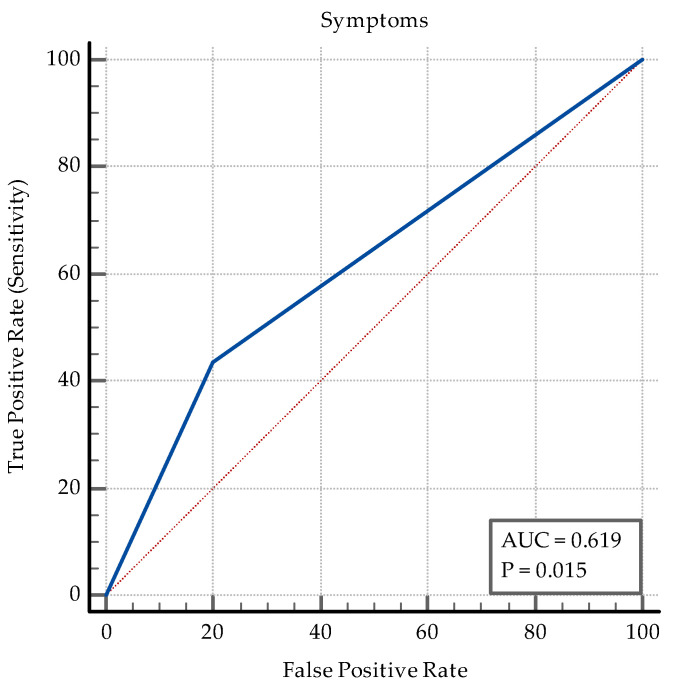
ROC curve for the severity of symptoms.

**Figure A9:**
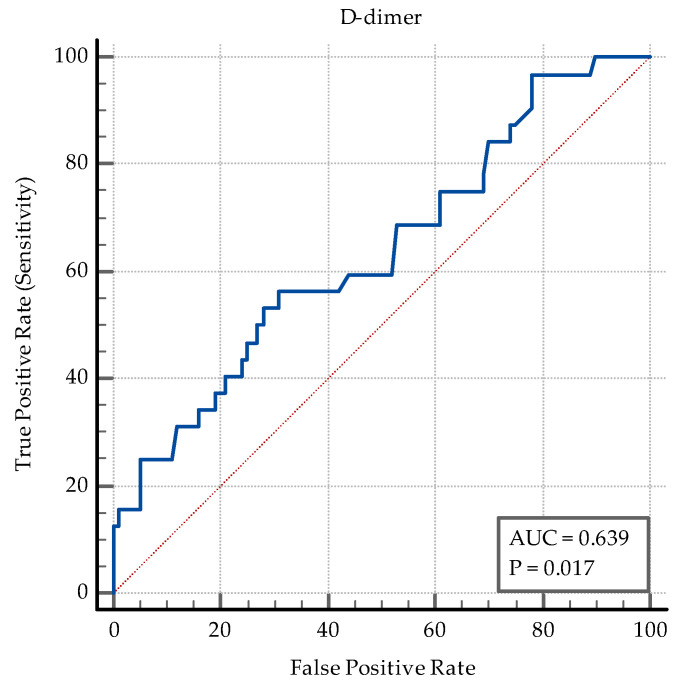
ROC curve for the value of D-dimer.

**Figure A10:**
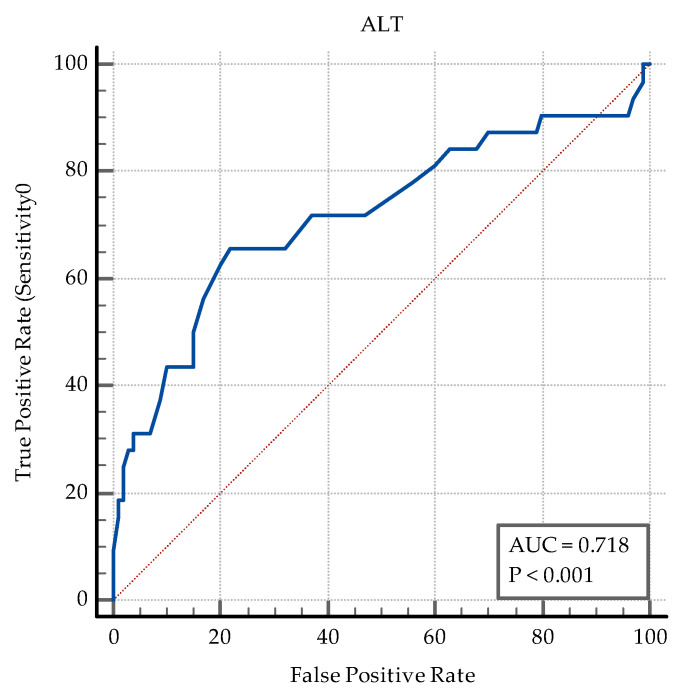
ROC curve for the value of ALT.

**Figure A11:**
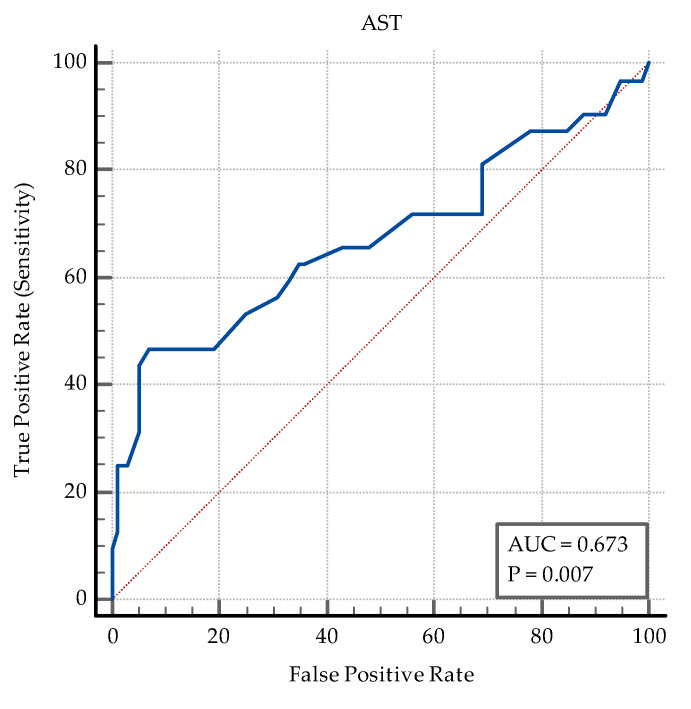
ROC curve for the value of AST.

Further on, the AUC-ROC analysis focused on clinical and biological parameters presented in Table 5. The following cut-off values were determined as significant for distinguishing between the presence and absence of PTB: SpO2 at diagnosis ≤89%, worst SpO2 ≤ 85%, CRP > 81 mg/L, IL-6 > 0.8 pg/mL, ALT > 35 U/L, AST > 40 U/L, D-dimer > 1.44 mg/L, lymphocyte count ≤ 2880/µL, PLR > 139.3, and the chest CT involvement score >14 (Figure 4 and Figure 5). The analysis revealed that SpO2 at diagnosis and the lowest SpO2 value have very good specificities, but relatively lower sensibilities. The gravity of the symptoms also has a good specificity, but quite a low sensibility. The CRP, ALT, AST and D-dimer levels revealed the same pattern. So do the imagistic parameters-chest X-ray and CT involvement score (Figure 5). In contrast, IL-6 and the lymphocytopenia revealed good sensibilities, with lower specificities (Table 5). See also Figure A6, Figure A7, Figure A8, Figure A9, Figure A10 and Figure A11.

Fisher’s exact test was used to evaluate the connection between associating PTB infection and several instances presented in Table 6. Having TB prior to the present episode and having a more severe symptomatology were revealed as significantly associated with the likelihood of associating a TB infection.

**Figure 6:**
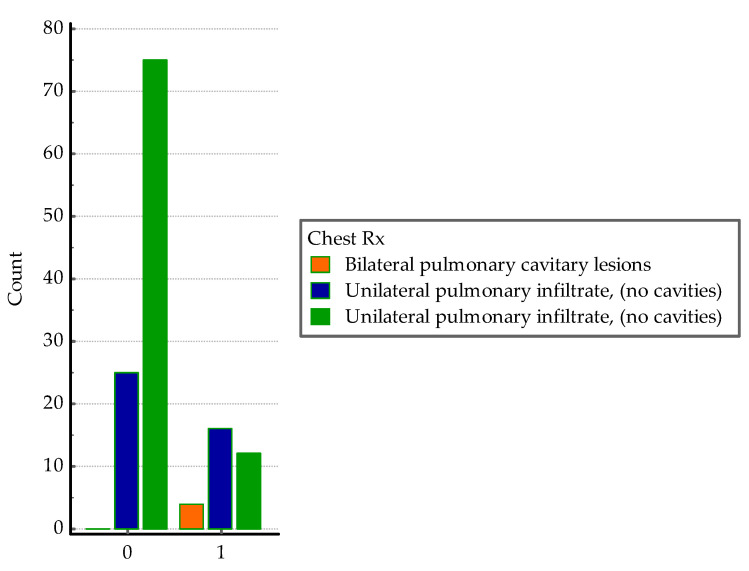
Distribution of X-ray pulmonary lesions in the two groups; 0 = *SARS-CoV-2* group, 1 = co-infection group.

The Chi-squared test was used to analyze associations between the imagistic lesions shown on chest X-ray and chest CT. The severity of the lesions on X-ray were significantly associated with co-infection (Chi-squared = 22.55, DF = 2, *p* < 0.0001), see Figure 6.

**Figure 7:**
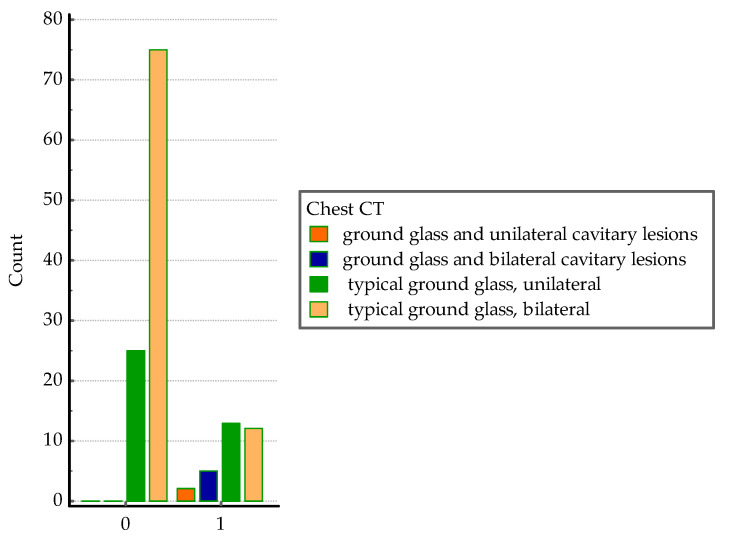
Distribution of CT pulmonary lesions in the two groups; 0 = *SARS-CoV-2* group, 1 = co-infection group.

With regard to the presence of ground glass opacities and cavitary lesions on chest CT, the following analysis involved only their presence. Ground glass and bilateral cavitary lesions were considered the most severe finds and were significantly associated with co-infection (Chi-squared = 29.1, DF = 3, *p* < 0.0001), see Figure 7.

However, according to the chest CT involvement score, we divided the subjects into two groups according to the previous AUC-ROC analysis result showing that a cut-off >14 is significant for the likelihood of co-infection, in our cohort (Table 7). 

Within the two categories of chest CT involvement score, there were no differences between coinfected and subjects with COVID-19 alone in neither >14 category (*p* = 0.8, Mann–Whitney test), nor ≤ 14 (*p* = 0.85, T-test).

**Figure 8:**
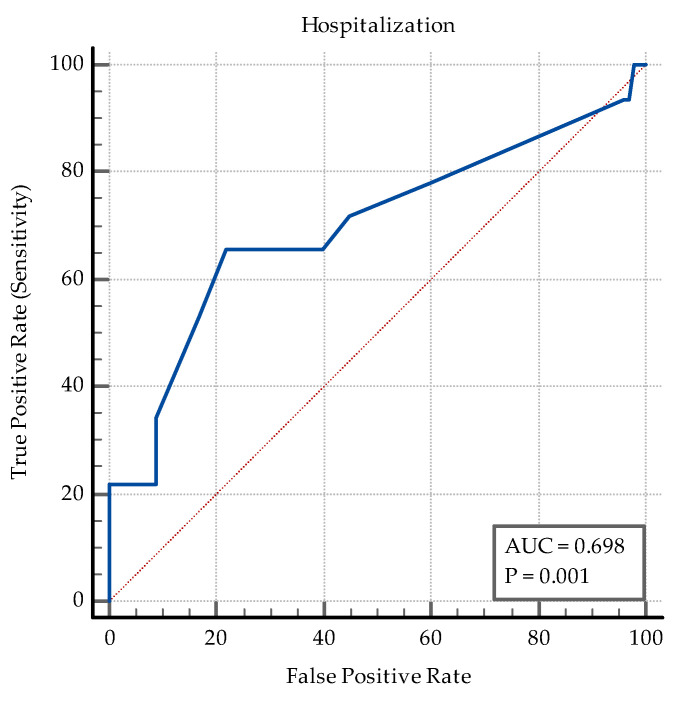
ROC curve for the days of hospitalization.

The length of hospitalization is also a reliable parameter that discerns between the two groups, with a cut-off (criterion) of >10 days, AUC = 0.69, *p* = 0.001, Se% = 65.62, 95% CI = 46.8–81.4, Sp% = 78, 95% CI = 68.6–85.7, PPV% = 48, NPV% = 87.6 (see Figure 8).

**Figure 9:**
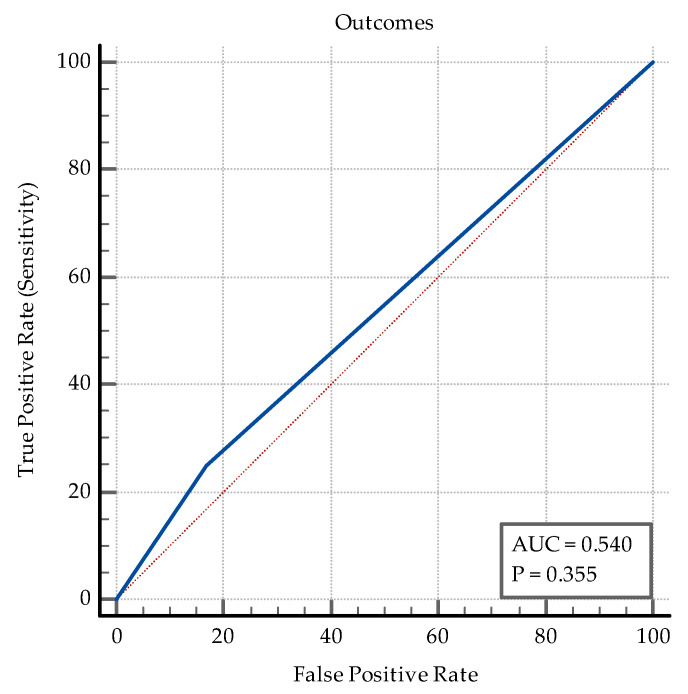
ROC curve for outcomes.

With regard fatal outcomes, 25% (*n* = 8) of the patients in the co-infection group and 17% in the *SARS-CoV-2* group died. The AUC-ROC analysis did not show that a fatal outcome is a reliable discriminator between the two instances (AUC = 0.54, *p* = 0.35, Se% = 25, 95% CI = 11.5–43.4, Sp% = 83, 95% CI = 74.2–89.8, PPV% = 32, NPV% = 76.6), see Figure 9. 

The logistic regression analysis did not retain the presence of PTB-*SARS-CoV-2* co-infection as a significant predictor of fatality. A multiple parameter logistic regression was employed to evaluate the effects of several markers (independent variables: severe symptoms, smoking, COPD, type 2 DM, prior TB infection) on outcome (dependent variable). The result showed that the presence of type 2 DM, severe symptomatology and longer hospitalization are significant independent predictors of fatal outcome (Table 8).

## 4. Discussion

This study aimed to analyze the impact of *SARS-CoV-2* infection on patients with PTB. To achieve that we compared two groups: one with PTB and *SARS-CoV-2* co-infection and a group with only *SARS-CoV-2* infection. The discussion will focus on the key findings and their implications in understanding the interaction between these two infectious diseases.

The demographic characteristics of the study population revealed a predominance of males in the co-infected group, with a 1.9:1 male to female ratio, whereas the *SARS-CoV-2* group had a more balanced sex distribution (52% males). Globally, tuberculosis affects a significantly higher number of men than women. The exact reason for this sex disparity remains uncertain, with epidemiological factors traditionally regarded as the primary drivers. One common explanation suggests that the male bias observed in TB cases stems from systematic underreporting and underdiagnosis of the disease in women [50]. A comprehensive meta-analysis of 29 surveys across 14 countries revealed a consistent male bias in both notification and prevalence rates [51], hence, considering the consistent reports about sex bias around the world, it is strongly suggested that biological sex differences do exist [52]. 

Furthermore, although age is a significant risk factor for both TB [53] and a more severe form of COVID-19 [54], this study did not detect significant differences in age between the two studied groups, due to the design of the study, which ensured that the selection of patients in the *SARS-CoV2* group is as similar as possible to the co-infection group. 

This study confirms lower BMI as a significant marker of a probable PTB-*SARS-CoV-2* co-infection. Low BMI represents a risk factor for developing lung TB due to an immunomodulatory effect on cytokine and chemokine response [55]. It has been known for decades that a BMI 10% lower than the ideal body weight increases the risk of developing lung TB three times in young men [56], and that the lowest BMI category is associated with a fivefold risk of lung TB compared to the highest BMI category [57], as shown in two very large cohort studies. The inverse link between BMI and the risk of developing lung TB has been reconfirmed multiple times [58,59,60]. In contrast, COVID-19 is associated with a higher risk of severe outcomes in overweight and obese individuals [61,62]. On the other hand, however, frailty, a common characteristic of TB patients, represents a major risk factor for mortality or longer hospitalization in COVID-19 patients [63]. Frailty is associated with both extremes of the BMI spectrum, and is often observed in both underweight [64] and severely obese individuals [65]. A healthy BMI may reduce the prevalence of frailty [64], leading to better outcomes in both acute and chronic ailments. Nevertheless, BMI is not a clinical marker with reliable specificity to TB infection, as numerous other pulmonary and extrapulmonary pathologies might associate weight loss. In situations where these pathologies are excluded, such as our study, a lower BMI could bring additional information for the diagnostic effort.

Social determinants such as poverty, living conditions, population density, and economic status play a role in influencing the incidence of COVID-19 and TB [66,67]. Employment status did not emerge as significantly impactful for the likelihood of co-infection in our study. However, the proportion of unemployed individuals reached 25% in the TB group, compared to 10% in the *SARS-CoV-2* group. Unemployment in TB patients poses a significant challenge, as they seem to exhibit more severe radiographic abnormalities and increased occurrences of treatment discontinuations and elevated mortality rates during hospitalization [68]. 

More than a third of both studied groups were smokers, but smoking did not emerge as a significant marker or predictor of co-infection. Smoking is connected to TB through its potential of damaging the immune response through defects in macrophages, monocytes and CD4 lymphocytes function, and hence, making the organism more susceptible to TB infection [69]. Most studies agree that smoking also increases the susceptibility to *SARS-CoV-2* infection, about 1.5 times according to a comprehensive literature review [64], although differences in populations and tobacco products (e-cigarettes) have been invoked as less harmful or even protective in a few studies [70]. A further impactful risk factor can be heavy alcohol consumption. Its toxic impact on the immune system heightens the risk of developing active illness. Alcohol intake stands as one of the foremost modifiable risk factors for tuberculosis, with alcohol use disorders prevalent in 30% of TB cases and contributing to 11.4% (9.3–13%) of TB-related mortality [71]. Regarding the subjects of our study, 90% declared no alcohol consumption at all, with the remaining occasionally consuming small amounts.

Comorbidities such as COPD and type 2 DM are aggravating factors for both TB and *SARS-CoV-2* infection. COPD is characterized by a combination of emphysema and chronic bronchitis, leading to chronic systemic inflammation that weakens the immune system. People with COPD are prone to various additional health problems, including heart failure, diabetes, atherosclerosis, osteoporosis, muscle loss, and co-infections. Among these complications, active TB poses a significant risk for individuals with COPD. Recent research indicates that individuals with COPD are three times more likely to develop active TB compared to those without. Moreover, once infected, individuals with COPD and TB face twice the risk of mortality compared to those without these conditions [72]. On the other hand, COPD is associated with worse outcomes in *SARS-CoV-2* infection, although data is unclear on whether COPD increases the susceptibility to coronavirus infection [73]. This study has not shown that COPD increases the probability of co-infection or the risk of fatal outcome. In contrast, associating type 2 DM has been significantly more prevalent in our study (59.4% of the subjects in the co-infection group presented type 2 DM, versus 18.7%, COPD). Diabetes heightens the susceptibility to TB and correlates with the manifestation of severe cavitating disease as well as unfavorable treatment outcomes, including mortality [74,75,76,77]. An underlying cause is the exacerbation of insulin resistance and stress-induced hyperglycemia by TB, which may ameliorate during the course of treatment [78,79]. It is even advisable to screen TB patients for DM after 2–3 months of initiating TB treatment [80]. Furthermore, diabetic patients with *SARS-CoV-2* infection are expected to present worse symptomatology and even worse outcomes. SpO2 at hospital admission, along with glycemia and glycosylated hemoglobin, seem to have the highest sensitivity and specificity in predicting the prognosis of type 2 DM patients with *SARS-CoV-2* infection [81]. Associating TB and type 2 DM in the context of *SARS-CoV-2* has not been studied sufficiently. Our study shows that type 2 DM increases the likelihood of a fatal outcome, but the analysis did not confirm it as significant for predicting co-infection. Further studies of this grave trio should be encouraged in larger samples. Another very significant comorbidity which was excluded from this particular study is represented by an impaired kidney function. Chronic kidney disease (CKD) can predispose individuals to TB due to impaired immune function and reduced ability to fight infections. In contrast, TB can exacerbate kidney function impairment in patients with CKD, leading to worsening renal outcomes. Additionally, CKD and COVID-19 are considered significant risk factors for severe COVID-19 outcomes. Patients with CKD often have underlying conditions such as diabetes and hypertension, which further increase their vulnerability to severe COVID-19. COVID-19 can directly affect kidney function through various mechanisms, including direct viral invasion of renal cells, systemic inflammation, and cytokine release syndrome. Acute kidney injury is a common complication of severe COVID-19 and can exacerbate pre-existing CKD or lead to new-onset kidney dysfunction [82].

A greater severity of acute symptomatology in COVID-19 at hospital admission is associated with a higher probability of co-infection with PTB [16,83,84,85]. In this regard, our study aligns with other findings. These results should be taken into consideration specifically in TB-endemic regions, especially in these post-pandemic times when *SARS-CoV-2* infection has become less concerning. Moreover, severe symptoms are also connected with higher odds of fatal outcome. 

In comparing patients with co-infection to those with *SARS-CoV-2* infection alone, significant differences emerge across various parameters. Co-infected individuals exhibited notably lower levels of SpO2 at both diagnosis and at their lowest recorded value, and higher levels of systolic blood pressure. The logistic regression connected lower SpO2 levels to a greater probability of co-infection. A systematic review of case reports of TB-COVID-19 co-infections has placed SpO2 levels at 90–97% room air in most cases [86]; the lower the SpO2, the worse the outcome [87]. Contrary to our results, the aforementioned review reported that about a third of the cases presented hypotension [86]. Among individuals with COVID-19 alone, arterial hypertension is considered a prevalent comorbidity. Due to the involvement of angiotensin converting enzyme 2 in *SARS-CoV-2* infection, there is speculation regarding the potential role of hypertension in the pathogenesis of COVID-19 [88]. Hypertension correlates with a 2.5-fold heightened risk of elevated disease severity and mortality among COVID-19 patients. Moreover, this association was predominantly observed among individuals aged over 60 years [89].

The present study reported diminished lymphocyte count and higher levels of C-reactive protein, transaminases, and D-dimer in the co-infection group. Lymphopenia is consistently identified as a characteristic observed in cases of COVID-19 [86,90,91,92]. It has been suggested that in addition to the specific clinical signs and symptoms, lymphopenia could be helpful in suspecting and isolating cases [90]. Neutrophilia is more typical in COVID-19, with severe neutrophilia being correlated to severe disease [93,94]. Our study reported normal median neutrophil count in the *SARS-CoV2* group, and significantly higher neutrophil levels in the co-infection group. Neutrophilia is a protective immune response in active TB infection, with neutrophil counts normalizing usually within 6 months of treatment [95]. Neutropenia is a rare complication of anti-tuberculous treatment, such as isoniazid [96]. Referring again to the previously mentioned review of case reports, the rise in serum inflammatory markers such as CRP, D-dimer, and IL-6 was reported to have a significant association with unfavorable outcome [86]. Moreover, when substantially elevated, these markers predict mortality and are used as a main indicator of intensive care need [97]. Liver enzymes are typically elevated due to activation of immune responses and represent markers of systemic inflammation [86,98]. Our study showed that higher ALT and neutrophil count levels are associated with increased odds of co-infection. With regard to the AUC-ROC analysis, parameters such as SpO2, CRP, ALT, AST, and D-dimer levels exhibited good discriminatory power between co-infected and COVID-19 patients alone, while others like IL-6 and lymphocyte count showed higher sensitivity but lower specificity.

Systemic inflammation was also analyzed via three surrogate markers: the neutrophil to lymphocyte ratio (NLR), the platelet to lymphocyte ratio (PLR), and the systemic immune-inflammation index (SII). These are markers usually utilized to determine the prognosis of viral or bacterial infections. They are considered more feasible, cost-effective, and accessible markers, that can be readily conducted within any healthcare facility setting [99,100].

High NLR accurately predicts disease severity and mortality in COVID-19 [101,102]. The pre-treatment NLR upon admission could serve as a valuable biomarker for predicting mortality and the onset of acute respiratory distress syndrome in individuals with miliary tuberculosis [103]. A recent meta-analysis has shown that NLR has a reliable power to discriminate between PTB infection and bacterial community acquired pneumonia—the lower the NLR, the higher the chances of PTB infection [104]. Our study has not showed significant differences in NLR between the two groups. 

Blood hypercoagulability is a prevalent condition observed among hospitalized COVID-19 patients, often accompanied by elevated D-dimers [105]. Thrombocytopenia has been associated with disease severity in several studies [106], while others have noted that patients with significantly elevated platelet counts tend to experience longer hospitalization stays. This latter observation is thought to be linked to the correlation between platelet count and the cytokine storm associated with *SARS-CoV-2* infection. Specifically, IL-6 promotes megakaryocyte generation by stimulating thrombopoietin levels, leading to elevated platelet counts [107]. The PLR, particularly during the peak of the platelet count, has emerged as an independent prognostic factor for prolonged hospitalization [108]. Moreover, an increased PLR is associated with increased risk of fatal outcome [108,109]. Elevated PLR levels have also been examined in several studies regarding TB. It has been shown to discriminate between TB infection and non-infection in COPD patients [110]. Our study showed that PLR is significantly higher in the case of PTB-*SARS-CoV-2* co-infected patients. Moreover, it showed that a PLR > 139.3 can distinguish between the two groups with a specificity of 79% (*p* = 0.04).

Like the NLR and PLR, the SII serves as a proinflammatory marker of systemic inflammation and holds potential for independently predicting mortality in COVID-19 cases [111]. SII is also a reliable marker of inflammation in TB patients [112]. Our study revealed more elevated SII in the co-infection group, but the difference was not statistically significant. Further studies should be employed on larger samples in order to clarify the role of these three surrogate markers in PTB-*SARS-CoV-2* co-infection.

Additionally, our study showed that the severity of lesions on chest X-ray and CT scans was significantly associated with co-infection, highlighting the importance of imaging modalities in diagnosis and prognosis. Chest X-rays are typically the initial imaging modality of choice due to their widespread availability and cost-effectiveness. However, chest CT scans offer greater sensitivity compared to conventional X-rays. They allow for the detection of complications beyond pulmonary involvement and can also indicate alternative diagnoses. In COVID-19 cases, the predominant radiological findings often include bilateral airspace opacities, such as consolidations and/or ground-glass opacities [113]. Imaging plays a crucial role in diagnosing and managing tuberculosis also. While chest X-rays serve as the primary imaging tool for pulmonary tuberculosis, CT is invaluable for evaluating both pulmonary and extrapulmonary manifestations of the disease [114]. Our study focused on the chest CT involvement score, which was significantly higher in co-infected subjects. Moreover, this score was shown to serve both as a significant independent predictor of co-infection, and as a significant discriminator between co-infection and COVID-19 alone, with a cut-off >14. One of the first studies to evaluate a PTB-*SARS-CoV-2* co-infected cohort showed that on chest CT evaluations, about 43% of patients presented multifocal ground-glass opacities distributed peripherally, specific to COVID-19, and about 47% presented lesions associated to TB such as cavitary lesions, branching micronodules, and consolidations [16]. Other studies have supported more or less the same findings, suggestive of both diseases [115,116].

The length of hospitalization emerged as a reliable parameter for distinguishing between the two groups, with a longer duration associated with co-infection. The analysis did not reveal a significant difference in fatal outcomes between the co-infected and *SARS-CoV-2* groups.

An observation on extrapulmonary TB and *SARS-CoV-2* co-infection is important, although our study did not explore this issue. Studies conducted during the same timeframe of the pandemic indicate a slight increase in extrapulmonary tuberculosis cases. The similarity in symptoms between pulmonary TB and COVID-19 may have skewed suspicions towards TB lung disease, potentially contributing to the uptick in extrapulmonary tuberculosis diagnoses [22]. Further multicenter studies involving larger populations from diverse regions would provide greater clarity on diagnosing extrapulmonary TB amidst the COVID-19 pandemic.

The key strengths of this study are the valuable results concerning a multitude of parameters which show that the initial premise that *SARS-CoV-2* has an additive impact on TB patients than on those TB-free, is true. Although a few studies with somewhat similar designs have been completed before and thus have been cited here, we believe our input is valuable to the global information on *PTB-SARS-CoV-2* co-infection, as TB infection is still an important issue in many parts of our world. We bring forward accessible and cost-efficient markers which can be used to determine the probability of co-infection in questionable situations. We consider the single-center nature of our study and thus, the sample size of the co-infection group as a limitation for this study, but, according to our pre-statistical estimations, the ratios were sufficient to provide statistical significance. Further research directions could explore extrapulmonary TB-COVID-19 co-infections or the impact of HIV or CKD on the outcome of TB-*SARS-CoV-2* co-infection. From an epidemiological point of view, the impact of anti-*SARS-CoV-2* vaccines on co-infection should be explored. Our study collected data from a period when vaccines were not available. However, vaccines have played a crucial role in slowing down and stopping the pandemic, and demonstrated significant efficacy in preventing severe forms of the disease and deaths associated with COVID-19. With regard to TB-*SARS-CoV-2* co-infection, further research is needed to better understand the duration of protection provided by vaccines, and the adaptation of vaccines to new virus strains [117].

## 5. Conclusions

The study highlights the intricate relationship between *SARS-CoV-2* infection and pulmonary tuberculosis, indicating that certain demographic, clinical, and biological factors may serve as potential indicators of TB co-infection in patients with *SARS-CoV-2*.

A lower BMI emerges as a significant marker suggesting underlying pulmonary TB in patients with *SARS-CoV-2*.

The presence of type 2 diabetes mellitus increases the likelihood of a fatal outcome in patients with PTB-*SARS-CoV-2* co-infection.

Co-infected patients exhibit more pronounced lymphocytopenia and higher levels of neutrophils, C-reactive protein, transaminases, D-dimer levels, and a higher chest CT involvement score.

High ALT and chest CT score are associated with an increased likelihood of co-infection. Parameters including SpO2, CRP, ALT, AST, D-dimer levels, and the chest CT score demonstrate good discriminatory power between co-infected individuals and those with COVID-19 alone. The platelet-to-lymphocyte ratio is notably elevated in co-infected patients.

While longer hospitalization durations are linked to co-infection, they do not significantly increase the likelihood of a fatal outcome.

## Figures and Tables

**Table 1:** Comparison between co-infected and *SARS-CoV-2* infected patients with regard to multiple parameters (Mann–Whitney test).

Parameter	Co-Infection Group Median Value	*SARS-CoV-2* group Median Value	*p*
SpO2 at diagnosis	90	94	**0.0009**
Lowest SpO2	83.5	89.5	**0.004**
Peripheral SBP at diagnosis	138	133	**0.04**
Peripheral DBP at diagnosis	92	87	0.07
CRP	89.5	55.5	**0.0001**
LDH	288	243.5	0.51
IL-6	4.2	1.2	0.08
AST	36	29	**0.003**
ALT	38.5	30	**0.0002**
D-dimer	1.91	0.91	**0.01**
Neutrophils *	6758.75 (SD = 3733.15)	5878.3 (SD = 1162.67)	**<0.001**
Lymphocytes	2210	3010	**0.003**
Thrombocytes	242.5 × 10^3^	252 × 10^3^	0.42
NLR	2	1.83	0.67
PLR	128.85	77.95	**0.03**
SII	538,198.71	459,783.69	0.7
Chest CT involvement score	16	12	0.002

* The T-student test was employed for neutrophil count. The bolded data in this table represents statistically significant results.

**Table 2:** Logistic regression depicting significant predictors of TB-*SARS-CoV-2* co-infection (part 1).

Parameter	Odds Ratio	95% CI	Coefficient	Std. Err.	Constant	*p*
BMI	0.76	0.65–0.89	−0.26	0.08	4.9	**0.001**
Symptoms severity	1.16	1.19–8.56	1.16	0.5	4.9	**0.02**

The bolded data in this table represents statistically significant results.

**Table 3:** Logistic regression depicting significant predictors of PTB-*SARS-CoV-2* co-infection (part 2).

Parameter	Odds Ratio	95% CI	Coefficient	Std. Err.	*p*
SpO2 at diagnosis	0.58	0.55–0.91	−0.33	0.12	**0.007**
LDH	0.98	0.98–0.99	−0.01	0.003	**0.0005**
ALT	1.05	1.01–1.11	0.05	0.02	**0.01**
Neutrophil count	0.99	0.99–1	−0.0002	0.0001	**0.03**
Chest CT involvement score	1.35	1.14–1.61	0.3	0.08	**0.0005**

The constant was 47.62. The bolded data in this table represents statistically significant results.

**Table 4:** AUC-ROC analysis for the clinical and anamnestic parameters, as discriminators between the presence and the absence of PTB co-infection in *SARS-CoV-2* patients (*n* = 132).

Parameter	AUC	*p*	Se%	95% CI	Sp%	95% CI	PPV %	NPV %
Age	0.6	0.058	81.25	63.6–92.8	39	29.4–49.3	29.9	87.7
Sex	0.58	0.08	68.75	50.0–83.9	48	37.9–58.2	29.7	82.8
BMI	**0.72**	**<0.0001**	84.37	67.2–94.7	67	56.9–76.1	43	93.1
Employment status	0.5	0.97	75	56.6–88.5	10	4.9–17.6	21.1	55.6
Smoking	0.51	0.72	37.5	21.1–56.3	66	55.8–75.2	26.1	76.7
COPD	0.5	0.97	81.25	63.6–92.8	19	11.8–28.1	24.3	76
Type 2 DM	0.59	0.052	37.5	21.1–56.3	81	71.9–88.2	38.7	80.2

The bolded data in this table represents statistically significant results.

**Table 5:** AUC-ROC analysis for the clinical, biologic and imagistic parameters, as discriminators between the presence and the absence of PTB co-infection in *SARS-CoV-2* patients (*n* = 132).

Parameter	AUC	*p*	Se%	95% CI	Sp%	95% CI	PPV %	NPV %
SpO2 at diagnosis	**0.69**	**0.002**	46.88	29.1–65.3	94	87.4–97.8	71.4	84.7
Lowest SpO2	**0.67**	**0.007**	62.5	43.7–78.9	72	62.1–80.5	41.7	85.7
Symptoms severity	**0.62**	**0.01**	43.75	26.4–62.3	80	70.8–87.3	41.2	81.6
CRP	**0.73**	**<0.001**	65.62	46.8–81.4	75	65.3–83.1	45.7	87.2
PCT	0.56	0.17	28.12	13.7–46.7	84	75.3–90.6	36	78.5
LDH	0.54	0.52	62.5	43.7–78.9	50	39.8–60.2	28.6	80.6
IL-6	**0.6**	**0.04**	81.25	63.6–92.8	45	35–55.3	32.1	88.2
AST	**0.67**	**0.006**	46.88	29.1–65.3	93	86.1–97.1	68.2	84.5
ALT	**0.72**	**0.0001**	65.62	46.8–81.4	78	68.6–85.7	48.8	87.6
D-dimer	**0.64**	**0.01**	56.25	37.7–73.6	69	59.0–77.9	36.7	83.1
Neutrophils	0.58	0.3	46.88	29.1–65.3	92	84.8–96.5	65.2	84.4
Lymphocytes	**0.67**	**0.002**	78.12	60–90.7	59	48.7–68.7	37.8	89.4
Thrombocytes	0.54	0.48	37.5	21.1–56.3	88	80–93.6	50	81.5
NLR	0.71	0.52	43.75	26.4–62.3	80	70.8–87.3	41.2	81.6
PLR	0.62	**0.04**	50	31.9–68.1	79	69.7–86.5	43.2	83.2
SII	0.52	0.74	43.75	26.4–62.3	76	66.4–84	36.8	86.9
Chest Rx	**0.7**	**<0.001**	62.5	43.7–78.9	75	65.3–83.1	44.4	86.2
Chest CT involvement score	**0.67**	**0.001**	62.5	43.7–78.9	73	63.2–81.4	42.6	85.9

The bolded data in this table represents statistically significant results.

**Table 6:** Associations between the probability of having TB co-infection and several parameters (Fisher’s exact test).

	Parameter	Fisher’s Exact Test *p*
Presence of PTB	Smoking	0.83
	COPD	0.99
	Type 2 DM	0.053
	TB prior to present episode	**<0.0001**
	Severe symptoms	**0.01**
	PCT	0.19
	Outcome	0.31

The bolded data in this table represents statistically significant results.

**Table 7:** Distribution of subjects according to the chest CT involvement score cut-off.

Chest CT Involvement Score	PTB-*SARS-CoV-2* Co-Infection *n*	Associated Cavitary Lesions *n*	*SARS-CoV-2* Co-Infection *n*
>14	20	2	27
≤14	12	2	73

**Table 8:** Logistic regression depicting significant predictors of fatal outcome.

Parameter	Odds Ratio	95% CI	Coefficient	Std. Err.	Constant	*p*
Type 2 DM	7.03	1.8–27.3	1.9	0.69	−4.78	**0.004**
Severe symptoms	12.17	3.1–47.75	2.49	0.69	−4.78	**0.0003**
Hospitalization	1.16	1.02–1.32	0.15	0.006	−4.78	**0.02**

The bolded data in this table represents statistically significant results.

## Data Availability

The findings of this study are supported by data which can be obtained upon request from the corresponding author. However, ethical restrictions prevent the public availability of the data.

## Abbreviations

ALT	Alanine aminotransferase
AST	Aspartate aminotransferase
AUC	Area under the receiver operating characteristic curve
BMI	Body mass index
COPD	Chronic Obstructive Pulmonary Disease
CRP	C-reactive protein
CT	Computer tomography
DM	Diabetes Mellitus
HIV	Human Deficiency Virus
IL-6	Interleukin 6
LDH	Lactate Dehydrogenase
*n*	number of subjects
*p*	*p*-value
PCT	Procalcitonin
PTB	pulmonary tuberculosis
ROC	Receiver operating characteristic
RT-PCR	Reverse Transcription Polymerase Chain Reaction
SpO2	Saturation of peripheral oxygen
TB	Tuberculosis
X-ray	high-energy electromagnetic radiation
WHO	World Health Organization

## References

[1] Mathiasen V.D., Andersen P.H., Johansen I.S., Lillebaek T., Wejse C. (2020). Clinical features of tuberculous lymphadenitis in a low-incidence country. Int. J. Infect. Dis..

[2] Visca D., Ong C.W.M., Tiberi S. (2021). Tuberculosis and COVID-19 interaction: A review of biological, clinical, and public health effects. Pulmonology.

[3] Wu Z., McGoogan J.M. (2020). Characteristics of and important lessons from the coronavirus disease 2019 (COVID-19) outbreak in China: Summary of a report of 72,314 cases from the Chinese Center for Disease Control and Prevention. JAMA.

[4] Chen N., Zhou M., Dong X., Qu J., Gong F., Han Y. (2020). Epidemiological and clinical characteristics of 99 cases of 2019 novel coronavirus pneumonia in Wuhan, China: A descriptive study. Lancet.

[5] Guan W.J., Ni Z.Y., Hu Y., Liang W.H., Ou C.Q., He J.X., China Medical Treatment Expert Group for COVID-19 (2020). Clinical Characteristics of Coronavirus Disease 2019 in China. N. Engl. J. Med..

[6] Ong C.W.M., Goletti D. (2020). Impact of the global COVID-19 outbreak on the management of other communicable diseases. Int. J. Tuberc. Lung Dis..

[7] Kames J., Holcomb D.D., Kimchi O., DiCuccio M., Hamasaki-Katagiri N., Wang T., Komar A.A., Alexaki A., Kimchi-Sarfaty C. (2020). Sequence analysis of SARS-CoV-2 genome reveals features important for vaccine design. Nat. Sci. Rep..

[8] Harvey W.T., Carabelli A.M., Jackson B., Gupta R.K., Thomson E.C., Harrison E.M., Ludden C., Reeve R., Rambaut A., COVID-19 Genomics UK (COG-UK) Consortium (2021). SARS-CoV-2 variants, spike mutations and immune escape. Nat. Rev. Microbiol..

[9] Farinholt T., Doddapaneni H., Qin X., Menon V., Meng Q., Metcalf G., Chao H., Gingras M.C., Farinholt P., Agrawal C. (2021). Transmission event of SARS-CoV-2 Delta variant reveals multiple vaccine breakthrough infections. medRxiv.

[10] Surleac M., Banica L., Casangiu C., Cotic M., Florea D., Sandulescu O., Milu P., Streinu-Cercel A., Vlaicu O., Paraskevis D. (2020). Molecular Epidemiology Analysis of SARS-CoV-2 Strains Circulating in Romania during the First Months of the Pandemic. Life.

[11] Lobiuc A., Dimian M., Gheorghita R., Sturdza O.A.C., Covasa M. (2021). Introduction and Characteristics of SARS-CoV-2 in North-East of Romania During the First COVID-19 Outbreak. Front. Microbiol..

[12] World Health Organization (2020). Global Tuberculosis Report 2020.

[13] Cilloni L., Fu H., Vesga J.F., Dowdy D., Pretorius C., Ahmedov S. (2020). The potential impact of the COVID-19 pandemic on the tuberculosis epidemic: A modelling analysis. EClinicalMedicine..

[14] Migliori G.B., Thong P.M., Akkerman O., Alffenaar J.W., Álvarez-Navascués F., Assao-Neino M.M. (2020). Worldwide Effects of Coronavirus Disease Pandemic on Tuberculosis Services, January–April 2020. Emerg. Infect. Dis..

[15] Buonsenso D., Iodice F., Sorba Biala J., Goletti D. (2021). COVID-19 effects on tuberculosis care in Sierra Leone. Pulmonology.

[16] Tadolini M., Codecasa L.R., García-García J.M., Blanc F.X., Borisov S., Alffenaar J.W. (2020). Active tuberculosis, sequelae and COVID-19 co-infection: First cohort of 49 cases. Eur. Respir. J..

[17] Mousquer G.T., Peres A., Fiegenbaum M. (2020). Pathology of TB/COVID-19 co-infection: The phantom menace. Tuberculosis.

[18] Singh A., Prasad R., Gupta A., Das K., Gupta N. (2020). Severe acute respiratory syndrome coronavirus-2 and pulmonary tuberculosis: Convergence can be fatal. Monaldi Arch. Chest Dis..

[19] Liao M., Liu Y., Yuan J., Wen Y., Xu G., Zhao J., Chen L., Li J., Wang X., Wang F. (2020). The landscape of lung bron-choalveolar immune cells in COVID-19 revealed by single-cell RNA sequencing. MedRxiv.

[20] Carlos W.G., Dela Cruz C.S., Cao B., Pasnick S., Jamil S. (2020). Novel Wuhan (2019-nCoV) Coronavirus. Am. J. Respir. Crit. Care Med..

[21] Rolo M., González-Blanco B., Reyes C.A., Rosillo N., López-Roa P. (2023). Epidemiology and factors associated with Extra-pulmonary tuberculosis in a Low-prevalence area. J. Clin. Tuberc. Other Mycobact. Dis..

[22] Udoakang A.J., Djomkam Zune A.L., Tapela K., Nganyewo N.N., Olisaka F.N., Anyigba C.A., Tawiah-Eshun S., Owusu I.A., Paemka L., Awandare G.A. (2023). The COVID-19, tuberculosis and HIV/AIDS: Ménage à Trois. Front. Immunol..

[23] Wang W., Xin C., Xiong Z., Yan X., Cai Y., Zhou K. (2020). Clinical characteristics and outcomes of 421 patients with coronavirus disease 2019 treated in a mobile cabin hospital. Chest.

[24] Wei M., Zhao Y., Qian Z., Yang B., Xi J., Wei J. (2020). Pneumonia caused by Mycobacterium tuberculosis. Microb. Infect..

[25] Narjess B., Faramarz M.J., Shabnam R. (2021). Mycobacterium tuberculosis and SARS-CoV-2 Coinfections: A Review. Front. Microbiol..

[26] Getnet F., Demissie M., Worku A., Gobena T., Tschopp R., Girmachew M. (2019). Delay in diagnosis of pulmonary tuberculosis increases the risk of pulmonary cavitation in pastoralist setting of Ethiopia. BMC Pulm. Med..

[27] Kaftan A.N., Hussain M.K., Algenabi A.A., Naser F.H., Enaya M.A. (2021). Predictive Value of C–reactive Protein, Lactate Dehydrogenase, Ferritin and D-dimer Levels in Diagnosing COVID-19 Patients: A Retrospective Study. Acta Inform. Med..

[28] Dheda K., Booth H., Huggett J.F., Johnson M.A., Zumla A., Rook G.A.W. (2005). Lung remodeling in pulmonary tuberculosis. J. Infect. Dis..

[29] Prakash A.K., Datta B., Goyal P., Chatterjee P., Gupta G. (2016). GENE-XPERT gives early diagnosis in early tuberculosis. Eur. Respir. J..

[30] COVID-19 Treatment Guidelines Panel Coronavirus Disease 2019 (COVID-19) Treatment Guidelines. National Institutes of Health. https://www.covid19treatmentguidelines.nih.gov/.

[31] Gounden V., Bhatt H., Jialal I. (2024). Renal Function Tests. [Updated 2023 Jul 17]. StatPearls.

[32] World Health Organization (2018). BCG vaccines: WHO position paper—February 2018. Wkly. Epidemiol. Rec..

[33] Purnell J.Q., Feingold K.R., Anawalt B., Blackman M.R., Boyce A., Chrousos G., Corpas E., de Herder W.W., Dhatariya K., Dungan K., Hofland J. (2000). Definitions, Classification, and Epidemiology of Obesity. [Updated 4 May 2023]. Endotext.

[34] Akpek M. (2022). Does COVID-19 Cause Hypertension?. Angiology.

[35] Parker C.S., Siracuse C.G., Litle V.R. (2018). Identifying lung cancer in patients with active pulmonary tuberculosis. JTD.

[36] Ramamoorthy S., Srinivas B.H., Badhe B.A., Jinkala S., Ganesh R.N. (2023). Coexistence of malignancy and tuberculosis: Is it double disease or double hit related to COVID-19?—Experience from a tertiary care center. Int. J. Clin. Exp. Pathol..

[37] Chung M.J., Goo J.M., Im J.-G. (2004). Pulmonary tuberculosis in patients with idiopathic pulmonary fibrosis. Eur. J. Radiol..

[38] Novikova L., Ilkovich Y., Speranskaya A. (2015). Tuberculosis in patients with idiopathic pulmonary fibrosis. Eur. Respir J..

[39] Metra M., Dinatolo E., Dasseni N. (2019). The New Heart Failure Association Definition of Advanced Heart Failure. Card. Fail. Rev..

[40] NHLBI Obesity Education Initiative Expert Panel on the Identification E and T of O in A (US), National Heart, Lung, and Blood Institute (1998). Clinical Guidelines on the Identification, Evaluation, and Treatment of Overweight and Obesity in Adults: The Evidence Report. Obesity Prevention and Management.

[41] Seifi S., Khatony A., Moradi G., Abdi A., Najafi F. (2018). Accuracy of pulse oximetry in detection of oxygen saturation in patients admitted to the intensive care unit of heart surgery: Comparison of finger, toe, forehead, and earlobe probes. BMC Nurs..

[42] Fukui S., Ikeda K., Kobayashi M., Nishida K., Yamada K., Horie S., Shimada Y., Miki H., Goto H., Hayashi K. (2023). Predictive prognostic biomarkers in patients with COVID 19 infection. Mol. Med. Rep..

[43] Ali E.T., Jabbar A.S., Al Ali H.S., Hamadi S.S., Jabir M.S., Albukhaty S. (2022). Extensive Study on Hematological, Immunological, Inflammatory Markers, and Biochemical Profile to Identify the Risk Factors in COVID-19 Patients. Int. J. Inflam.

[44] Ravindra R., Ramamurthy P., Aslam S.S.M., Kulkarni A., Suhail K., Ramamurthy P.S. (2022). Platelet Indices and Platelet to Lymphocyte Ratio (PLR) as Markers for Predicting COVID-19 Infection Severity. Cureus.

[45] Mangoni A.A., Zinellu A. (2023). Systemic inflammation index, disease severity, and mortality in patients with COVID-19: A systematic review and meta-analysis. Front. Immunol..

[46] Hansell D.M., Bankier A.A., MacMahon H., McLoud T.C., Müller N.L., Remy J. (2008). Fleischner society: Glossary of terms for thoracic imaging. Radiology.

[47] Hernandez-Romieu A.C., Little B.P., Bernheim A., Schechter M.C., Ray S.M., Bizune D., Kempker R. (2019). Increasing Number and Volume of Cavitary Lesions on Chest Computed Tomography Are Associated With Prolonged Time to Culture Conversion in Pulmonary Tuberculosis. Open Forum Infect Dis..

[48] Chang Y.-C., Yu C.-J., Chang S.-C., Galvin J.R., Liu H.-M., Hsiao C.-H., Kuo P.-H., Chen K.-Y., Franks T.J., Huang K.-M. (2005). Pulmonary sequelae in convalescent patients after severe acute respiratory syndrome: Evaluation with thin-section CT. Radiology.

[49] Yazdi N.A., Ghadery A.H., SeyedAlinaghi S., Jafari F., Jafari S., Hasannezad M., Koochak H.E., Salehi M., Manshadi S.A.D., Meidani M. (2021). Predictors of the chest CT score in COVID-19 patients: A cross-sectional study. Virol. J..

[50] Nhamoyebonde S., Leslie A. (2014). Biological Differences Between the Sexes and Susceptibility to Tuberculosis. J. Infect. Dis..

[51] Borgdorff M.W., Nagelkerke N.J., Dye C., Nunn P. (2000). Gender and tuberculosis: A comparison of prevalence surveys with notification data to explore sex differences in case detection. Int. J. Tuberc. Lung Dis..

[52] Min J., Park J.S., Kim H.W., Ko Y., Oh J.Y., Jeong Y.-J., Na J.O., Kwon S.-J., Choe K.H., Lee W.-Y. (2023). Differential effects of sex on tuberculosis location and severity across the lifespan. Sci. Rep..

[53] Caraux-Paz P., Diamantis S., de Wazières B., Gallien S. (2021). Tuberculosis in the Elderly. J. Clin. Med..

[54] Bartleson J.M., Radenkovic D., Covarrubias A.J., Furman D., Winer D.A., Verdin E. (2021). SARS-CoV-2, COVID-19 and the Ageing Immune System. Nat. Aging.

[55] Nathella Pavan K., Arul P.N., Kadar M., Pradeep A.M., Vaithilingam V.B., Nair D., Sujatha N., Babu S. (2023). Low Body Mass Index Is Associated with Diminished Plasma Cytokines and Chemokines in Both Active and Latent Tuberculosis. Front. Nutr..

[56] Edwards L.B., Livesay V.T., Acquaviva F.A., Palmer C.E. (1971). Height, Weight, Tuberculous Infection, and Tuberculous Disease. Arch. Environ. Health.

[57] Tverdal A. (1986). Body Mass Index and Incidence of Tuberculosis. Eur. J. Respir. Dis..

[58] Cho S.H., Lee H., Kwon H., Shin D.W., Joh H.-K., Han K., Park J.H., Cho B. (2022). Association of Underweight Status with the Risk of Tuberculosis: A Nationwide Population-Based Cohort Study. Sci. Rep..

[59] Choi H., Yoo J.E., Han K., Choi W., Rhee S.Y., Lee H., Shin D.W. (2021). Body Mass Index, Diabetes, and Risk of Tuberculosis: A Retrospective Cohort Study. Front. Nutr..

[60] Casha A.R., Scarci M. (2017). The Link between Tuberculosis and Body Mass Index. J. Pers. Med..

[61] Gao M., Piernas C., Astbury N.M., Hippisley-Cox J., O’Rahilly S., Aveyard P., Jebb S.A. (2021). Associations between Body-Mass Index and COVID-19 Severity in 6·9 Million People in England: A Prospective, Community-Based, Cohort Study. Lancet Diabetes Endocrinol..

[62] Lockhart S.M., O’Rahilly S. (2020). When Two Pandemics Meet: Why Is Obesity Associated with Increased COVID-19 Mortality?. Med.

[63] Hewitt J., Carter B., Vilches-Moraga A., Quinn T.J., Braude P., Verduri A., Pearce L., Stechman M., Short R., Price A. (2020). The Effect of Frailty on Survival in Patients with COVID-19 (COPE): A Multicentre, European, Observational Cohort Study. Lancet Public Health.

[64] Jayanama K., Theou O., Godin J., Mayo A., Cahill L., Rockwood K. (2022). Relationship of Body Mass Index with Frailty and All-Cause Mortality Among Middle-Aged and Older Adults. BMC Med..

[65] Watanabe D., Yoshida T., Watanabe Y., Yamada Y., Kimura M. (2020). A U-Shaped Relationship between the Prevalence of Frailty and Body Mass Index in Community-Dwelling Japanese Older Adults: The Kyoto–Kameoka Study. J. Clin. Med..

[66] Koupaei M., Naimi A., Moafi N., Mohammadi P., Tabatabaei F.S., Ghazizadeh S., Heidary M., Khoshnood S. (2021). Clinical Characteristics, Diagnosis, Treatment, and Mortality Rate of TB/COVID-19 Coinfected Patients: A Systematic Review. Front. Med..

[67] Duarte R., Aguiar A., Pinto M., Furtado I., Tiberi S., Lönnroth K., Migliori G.B. (2021). Different Disease, Same Challenges: Social Determinants of Tuberculosis and COVID-19. Pulmonology.

[68] Przybylski G., Dabrowska A., Pilaczyńska-Cemel M., Krawiecka D. (2014). Unemployment in TB Patients—Ten-Year Observation at Regional Center of Pulmonology in Bydgoszcz, Poland. Med. Sci. Monit..

[69] Altet M., Alcaide J., Plans P., Taberner J., Saltó E., Folguera L., Salleras L. (1996). Passive Smoking and Risk of Pulmonary Tuberculosis in Children Immediately Following Infection. A Case-Control Study. Tuberc. Lung Dis..

[70] Chattopadhyay S., Malayil L., Kaukab S., Merenstein Z., Sapkota A.R. (2023). The Predisposition of Smokers to COVID-19 Infection: A Mini-Review of Global Perspectives. Heliyon.

[71] Morojele N.K., Shenoi S.V., Shuper P.A., Braithwaite R.S., Rehm J. (2021). Alcohol Use and the Risk of Communicable Diseases. Nutrients.

[72] Inghammar M., Ekbom A., Engström G., Ljungberg B., Romanus V., Lofdahl C.G., Egesten A. (2010). COPD and the Risk of Tuberculosis—A Population-Based Cohort Study. PLoS ONE.

[73] Awatade N.T., Wark P.A.B., Chan A.S.L., Mamun S.M.A.A., Mohd Esa N.Y., Matsunaga K., Rhee C.K., Hansbro P.M., Sohal S.S. (2023). The Complex Association between COPD and COVID-19. J. Clin. Med..

[74] Shewade H.D., Jeyashree K., Mahajan P., Shah A.N., Kirubakaran R., Rao R., Kumar A.M.V. (2017). Effect of Glycemic Control and Type of Diabetes Treatment on Unsuccessful TB Treatment Outcomes among People with TB-Diabetes: A Systematic Review. PLoS ONE.

[75] Baker M.A., Harries A.D., Jeon C.Y., Hart J.E., Kapur A., Lönnroth K., Ottmani S.-E., Goonesekera S.D., Murray M.B. (2011). The Impact of Diabetes on Tuberculosis Treatment Outcomes: A Systematic Review. BMC Med..

[76] Faurholt-Jepsen D., Range N., PrayGod G., Jeremiah K., Faurholt-Jepsen M., Aabye M.G., Changalucha J., Christensen D.L., Grewal H.M.S., Martinussen T. (2013). Diabetes is a Strong Predictor of Mortality during Tuberculosis Treatment: A Prospective Cohort Study among Tuberculosis Patients from Mwanza, Tanzania. Trop. Med. Int. Health.

[77] Moreira J., Castro R., Lamas C., Ribeiro S., Grinsztejn B., Veloso V.G. (2018). Hyperglycemia during Tuberculosis Treatment Increases Morbidity and Mortality in a Contemporary Cohort of HIV-Infected Patients in Rio de Janeiro, Brazil. Int. J. Infect. Dis..

[78] Dungan K.M., Braithwaite S.S., Preiser J.C. (2009). Stress Hyperglycaemia. Lancet.

[79] Kubjane M., Berkowitz N., Goliath R., Levitt N.S., Wilkinson R.J., Oni T. (2020). Tuberculosis, HIV and the Association with Transient Hyperglycaemia in Peri-Urban South Africa. Clin. Infect. Dis..

[80] Ottmani S.E., Murray M.B., Jeon C.Y., Baker M.A., Kapur A., Lönnroth K., Harries A.D. (2010). Consultation Meeting on Tuberculosis and Diabetes Mellitus: Meeting Summary and Recommendations. Int. J. Tuberc. Lung Dis..

[81] Albai O., Braha A., Timar B., Sima A., Deaconu L., Timar R. (2024). Assessment for Clinical Outcome in Patient with SARS-CoV-2 Infection and Diabetes Mellitus. Diabetes Metab. Syndr. Obes..

[82] Mathur S.B., Saxena R., Pallavi P., Jain R., Mishra D., Jhamb U. (2022). Effect of Concomitant Tuberculosis Infection on COVID-19 Disease in Children: A Matched, Retrospective Cohort Study. J. Trop. Pediatr..

[83] Habib G., Mahmood K., Ahmad L., Gul H., Hayat A., Ur Rehman M. (2023). Clinical Manifestations of Active Tuberculosis Patients Coinfected with Severe Acute Respiratory Syndrome Coronavirus-2. J. Clin. Tuberc. Other Mycobact. Dis..

[84] Daneshvar P., Hajikhani B., Sameni F., Noorisepehr N., Zare F., Bostanshirin N., Yazdani S., Goudarzi M., Sayyari S., Dadashi M. (2023). COVID-19 and Tuberculosis Coinfection: An Overview of Case Reports/Case Series and Meta-Analysis of Prevalence Studies. Heliyon.

[85] Gou J., Zhang G. (2022). Characteristics of COVID-19 and Tuberculosis Co-Infection: A Cross-Sectional Study in Henan Province. J. Clin. Med. Img..

[86] Mollalign H., Chala D., Beyene D. (2022). Clinical Features and Treatment Outcome of Coronavirus and Tuberculosis Co-Infected Patients: A Systematic Review of Case Reports. Infect. Drug Resist..

[87] Illg Z., Muller G., Mueller M., Nippert J., Allen B. (2021). Analysis of the Absolute Lymphocyte Count in COVID-19 Patients. Am. J. Emerg. Med..

[88] Tadic M., Cuspidi C., Grassi G., Mancia G. (2020). COVID-19 and Arterial Hypertension: Hypothesis or Evidence?. J. Clin. Hypertens..

[89] Lippi G., Wong J., Henry B.M. (2020). Hypertension in Patients with Coronavirus Disease 2019 (COVID-19): A Pooled Analysis. Pol. Arch. Intern. Med..

[90] Härter G., Spinner C.D., Roider J., Bickel M., Krznaric I., Grunwald S., Schabaz F., Gillor D., Postel N., Mueller M.C. (2020). A Case Series of 33 Patients with COVID-19 in People Living with Human Immunodeficiency Virus. Infection.

[91] Huang I., Pranata R. (2020). Lymphopenia in Severe Coronavirus Disease-2019 (COVID-19): A Systematic Review and Meta-Analysis. J. Intensive Care.

[92] Anai M., Akaike K., Iwagoe H., Akasaka T., Higuchi T., Miyazaki A., Naito D., Tajima Y., Takahashi H., Komatsu T. (2021). A Decrease in Hemoglobin Levels Predicts an Increased Risk for Severe Respiratory Failure in COVID-19 Patients with Pneumonia. Respir. Res..

[93] Li J., Zhang K., Zhang Y., Gu Z., Huang C. (2023). Neutrophils in COVID-19: Recent Insights and Advances. Virol. J..

[94] Janiuk K., Jabłońska E., Garley M. (2021). Significance of NETs Formation in COVID-19. Cells.

[95] Moideen K., Kumar N.P., Nair D., Banurekha V.V., Bethunaickan R., Babu S. (2018). Heightened Systemic Levels of Neutrophil and Eosinophil Granular Proteins in Pulmonary Tuberculosis and Reversal following Treatment. Infect. Immun..

[96] Cormican L.J., Schey S., Milburn H.J. (2004). G-CSF Enables Completion of Tuberculosis Therapy Associated with Iatrogenic Neutropenia. Eur. Respir. J..

[97] Noor F.M., Islam M.M. (2020). Prevalence and Associated Risk Factors of Mortality Among COVID-19 Patients: A Meta-Analysis. J. Commun. Health.

[98] Baj J., Karakuła-Juchnowicz H., Teresiński G., Buszewicz G., Ciesielka M., Sitarz R., Forma A., Karakuła K., Flieger W., Portincasa P. (2020). Specific and Non-Specific Clinical Manifestations and Symptoms: The Current State of Knowledge. J. Clin. Med..

[99] Zulfic Z., Weickert C.S., Weickert T.W., Liu D., Myles N., Galletly C. (2020). Neutrophil-Lymphocyte Ratio—A Simple, Accessible Measure of Inflammation, Morbidity and Prognosis in Psychiatric Disorders?. Australas. Psychiatry.

[100] Li Q., Xie J., Huang Y., Liu S., Guo F., Liu L., Yang Y. (2021). Leukocyte Kinetics During the Early Stage Acts as a Prognostic Marker in Patients with Septic Shock in Intensive Care Unit. Medicine.

[101] Sumardi U., Valentino B., Prasetya D., Debora J., Sugianli A.K. (2023). The Diagnostic Value of Kinetics of NLR to Identify Secondary Pulmonary Bacterial Infection Among COVID-19 Patients at Single Tertiary Hospital in Indonesia. Int. J. Gen. Med..

[102] Toori K.U., Qureshi M.A., Chaudhry A., Safdar M.F. (2021). Neutrophil to Lymphocyte Ratio (NLR) in COVID-19: A Cheap Prognostic Marker in a Resource Constraint Setting. Pak. J. Med. Sci..

[103] Han Y., Kim S.J., Lee S.H., Sim Y.S., Ryu Y.J., Chang J.H., Shim S.S., Kim Y., Lee J.H. (2018). High Blood Neutrophil-Lymphocyte Ratio Associated with Poor Outcomes in Miliary Tuberculosis. J. Thorac. Dis..

[104] Shojaan H., Kalami N., Ghasempour Alamdari M., Alorizy S.M.E., Ghaedi A., Bazrgar A., Khanzadeh M., Lucke-Wold B., Khanzadeh S. (2023). Diagnostic Value of the Neutrophil Lymphocyte Ratio in Discrimination between Tuberculosis and Bacterial Community Acquired Pneumonia: A Meta-Analysis. J. Clin. Tuberc. Other Mycobact. Dis..

[105] Connors J.M., Levy J.H. (2020). Thromboinflammation and the Hypercoagulability of COVID-19. J. Thromb. Haemost..

[106] Lippi G., Plebani M., Henry B.M. (2020). Thrombocytopenia is Associated with Severe Coronavirus Disease 2019 (COVID-19) Infections: A Meta-Analysis. Clin. Chim. Acta.

[107] Qu R., Ling Y., Zhang Y.H., Wei L.Y., Chen X., Li X.M., Liu X.Y., Liu H.M., Guo Z., Ren H. (2020). Platelet-to-Lymphocyte Ratio is Associated with Prognosis in Patients with Coronavirus Disease-19. J. Med. Virol..

[108] Urbano M., Costa E., Geraldes C. (2022). Hematological Changes in SARS-CoV-2 Positive Patients. Hematol. Transfus. Cell Ther..

[109] Simon P., Le Borgne P., Lefevbre F., Cipolat L., Remillon A., Dib C., Hoffmann M., Gardeur I., Sabah J., Kepka S. (2022). Platelet-to-Lymphocyte Ratio (PLR) Is Not a Predicting Marker of Severity but of Mortality in COVID-19 Patients Admitted to the Emergency Department: A Retrospective Multicenter Study. J. Clin. Med..

[110] Chen G., Wu C., Luo Z., Teng Y., Mao S. (2016). Platelet-Lymphocyte Ratios: A Potential Marker for Pulmonary Tuberculosis Diagnosis in COPD Patients. Int. J. Chron. Obstruct. Pulmon. Dis..

[111] Karaaslan T., Karaaslan E. (2022). Predictive Value of Systemic Immune-Inflammation Index in Determining Mortality in COVID-19 Patients. J. Crit. Care Med..

[112] Ştefanescu S., Cocoş R., Turcu-Stiolica A., Mahler B., Meca A.-D., Giura A.M.C., Bogdan M., Shelby E.-S., Zamfirescu G., Pisoschi C.-G. (2021). Evaluation of Prognostic Significance of Hematological Profiles After the Intensive Phase Treatment in Pulmonary Tuberculosis Patients from Romania. PLoS ONE.

[113] Martínez Chamorro E., Díez Tascón A., Ibáñez Sanza L., Ossaba Vélez S., Borruel Nacente S. (2020). Radiologic Diagnosis of Patients with COVID-19. Radiología Engl. Ed..

[114] Bomanji J.B., Gupta N., Gulati P., Das C.J. (2015). Imaging in Tuberculosis. Cold Spring Harb. Perspect. Med..

[115] Tham S.M., Lim W.Y., Lee C.K., Loh J., Premkumar A., Yan B., Kee A., Chai L., Tambyah P.A., Yan G. (2020). Four Patients with COVID-19 and Tuberculosis, Singapore, April-May 2020. Emerg. Infect. Dis..

[116] Mançano A.D., Zanetti G., Marchiori E. (2022). Concomitant COVID-19 and Pulmonary Tuberculosis: Computed Tomography Aspects. Radiol. Bras..

[117] Chirico F., Teixeira da Silva J.A., Tsigaris P., Sharun K. (2022). Safety & Effectiveness of COVID-19 Vaccines: A Narrative Review. Indian J. Med. Res..

